# Inflammatory Markers in Substance Use and Mood Disorders: A Neuroimaging Perspective

**DOI:** 10.3389/fpsyt.2022.863734

**Published:** 2022-04-26

**Authors:** Khushbu Agarwal, Peter Manza, Marquis Chapman, Nafisa Nawal, Erin Biesecker, Katherine McPherson, Evan Dennis, Allison Johnson, Nora D. Volkow, Paule V. Joseph

**Affiliations:** ^1^Section of Sensory Science and Metabolism Unit, Division of Intramural Research, Department of Health and Human Services, National Institutes of Health, National Institute on Alcohol Abuse and Alcoholism, Bethesda, MD, United States; ^2^Section of Sensory Science and Metabolism, Division of Intramural Research, U.S. Department of Health and Human Services, National Institute of Nursing Research, National Institutes of Health, Bethesda, MD, United States; ^3^Laboratory of Neuroimaging, Department of Health and Human Services, National Institute on Alcohol Abuse and Alcoholism, Bethesda, MD, United States

**Keywords:** addiction, bipolar disorder (BD), major depressive disorder (MDD), inflammation, positron emission tomography (PET), functional magnetic resonance imaging (fMRI), magnetic resonance spectroscopy or MRS

## Abstract

Chronic exposure to addictive drugs in substance use disorders and stressors in mood disorders render the brain more vulnerable to inflammation. Inflammation in the brain, or neuroinflammation, is characterized by gliosis, microglial activation, and sustained release of cytokines, chemokines, and pro-inflammatory factors compromising the permeability of the blood-brain barrier. There is increased curiosity in understanding how substance misuse and/or repeated stress exposure affect inflammation and contribute to abnormal neuronal activity, altered neuroplasticity, and impaired cognitive control, which eventually promote compulsive drug-use behaviors and worsen mood disorders. This review will emphasize human imaging studies to explore the link between brain function and peripheral markers of inflammation in substance use disorders and mood disorders.

## Background

Substance Use Disorders (SUDs) and associated comorbidities like mood disorders have increased public health concern significantly. The rise in SUD cases have had substantial economic burden in the United States (annual cost of $740 billion) pertaining to health care, reduction in productivity, as well as increased crime rates (1). A major hindrance to treatment is posed by the presence of comorbid conditions like depressive and anxiety symptoms (2). Moreover, due to the high co-occurrence of mood disorders, some of the boundaries between clinical presentations of SUD and mood disorders remains unclear. SUD individuals may present symptoms mirroring those suffering from affective mood disorders. The mood disorders are either alleviated or exacerbated by current use or withdrawal of substance of choice, respectively. Clinicians and researchers formulated a set of diagnostic criteria to help differentiate whether patients fall within the domain of SUD or Mood disorders. This requires a thorough assessment of a patients’ history of mood disorders in conjunction with a battery of questionnaires assessing the possibility of substance overuse (3, 4).

Dysfunction of the immune system and chronic inflammation have been implicated in the pathophysiology of substance use and related psychiatric disorders (5–7), and understanding this dysfunction may help understand the differences and similarities of mood disorders and SUDs. Inflammatory responses triggered by the substance of abuse and/or any stressor stimuli are mediated by cytokines, and this group includes interleukins (ILs), chemokines, interferons (IFNs), tumor necrosis factors (TNF), and lymphokines. These cytokines are generated by cells of the immune system, like macrophages, lymphocytes (T-cells, B-cells, and natural killer cells), also non-immune cells, including some stromal, endothelial cells, and fibroblasts. To modulate the immune response, the cytokines act through receptors and enhance the immune response or show an anti-inflammatory effect by affecting various biological processes (8). Neuroinflammation is thought to contribute to the neural adaptations following chronic exposure to addictive substances, including illicit drugs (6), as well as stressors in mood disorders (9).

The neuroimmune response induced by addictive substances (alcohol, nicotine, and illicit drugs) to an extent is characterized by proliferation, morphological and functional changes of microglia (10). Microglial cells are distributed throughout the brain and abundantly in substantia nigra, basal ganglia, and hippocampus (11). Microglia respond directly to substance-induced central nervous system (CNS) injury. They are activated by stimulating chemokine and cytokine receptors or peripheral signals, potentially resulting from substance-induced damage to the blood-brain barrier (BBB) (6). The activated microglia initiate downstream processes involving cell migration to the site of injury and phagocytosis (12), the production of pro-inflammatory factors, such as IL-1β, IL-6, and TNF-α, and the generation of reactive oxygen (ROS) and nitrogen species that cause neuronal damage (13). Thus, its noted that excessive release of neurotransmitters due to drug use may bind to receptors expressed on glial cells and further amplify inflammatory signaling *via* additional release of cytokines and chemokines, potentially contributing to the positive feedback that promotes inflammation.

Dysregulation of neuroimmune signaling increases neurotoxicity which augments neurodegenerative processes, and disrupts neuronal function, exacerbating drug-related behaviors and comorbid mood disorders (6, 14). The neurodegerative processes manifest as loss of neuronal and glial cells and the typical form of presentations include: clinical features like dementia, parkinsonism, or motor neuron disease, and/or anatomically as frontotemporal degenerations, extrapyramidal disorders, or spinocerebellar degeneration (15). In opioid users, degeneration of the white matter known as toxic leukoencephalopathy causes inattention, personality changes, dysarthria, ataxia, dementia, coma and even death (16). Also in chronic opioid users neurodegeneration may lead to ubiquitin-positive neurons (17). Young chronic heroin users showed an enhancement in the count and distribution of hyperphosphorylated tau-positive neurofibrillary pre-tangles (17). Moreover, heroin users often reveal lesions in the globus pallidus due to hypoxic injury caused by excessive drug usage (18). Neurodegeneration in chronic excessive alcohol drinkers is mainly initiated by oxidative stress and is presented as cognitive impairment due to structural abnormalities in white matter, axonal loss and demyelination in hippocampus, frontal lobe, and corpus callosum [detailed in review by Kamal et al. (19)]. It has been well documented that depression is associated with neurodegenerative diseases like Alzheimer’s, Parkinson’s, or Huntington’s disease, while some hypothesize that occurrence of depression itself indicates the presence of latent neurodegeneration (20). Neurodegenerative outcomes like argyrophilic grain-type tauopathy and Lewy body-related alpha-synucleinopathy were reported in a case study on bipolar disorder (21). The interaction between neuroinflammation, neurodegeneration, and bipolar and major depressive disorders is detailed in reviews by Brown et al. (20), Hurley and Tizabi (22), and Serafini et al. (23).

Although the neural circuits relevant to substance misuse and mood disorders may be impaired before inflammation, activation of microglia and other glia-mediated synaptic remodeling induced by drug/stressors may further compromise brain function in these individuals. For example, many addictive substances/stressors render the brain more vulnerable to inflammation and contribute to neuropathology (14, 24). Genomic study on brain tissue samples from MDD and BD patients revealed increased mRNA expression of inflammatory genes in frontal region, reward related brain region (25, 26). Furthermore, epigenome-wide association studies (EWAS) have revealed epigenetic associations between inflammatory genes like GAS5 and phenotypes such as fear conditioning and extinction in chronic alcohol drinkers (27). These studies provide a basis for the transcriptional regulation of neuroendophenotypes in mood and substance use disorders by inflammatory genes.

Understanding how substance misuse and/or repeated stress exposure associated with inflammation may contribute to aberrant neuronal activity, altered neuroplasticity, impair cognitive control, eventually promote compulsive drug-use behaviors, and worsen mood disorders has gained immense interest over time. This review will highlight research conducted using imaging techniques linking brain function to peripheral markers of inflammation in substance use disorders and mood disorders.

### Microglial Activation and Translocator Protein Levels in Brain Regions of Substance Misusers

Substance misuse disrupts the brain’s neuroimmune system such that microglial counts increase, leading to the release of pro-inflammatory cytokines like TNF-α, and IL-1β, which may eventually cause neuronal dysfunction and death (14). Microglia are critical players in the immune surveillance system of the central nervous system, in which they act as macrophages and are first responders to brain insults (28). Following the initial response, microglia are transformed from a sentry state into an active state and increase the expression of an outer mitochondrial membrane protein, the 18-kDa translocator protein (TSPO). Thus, TSPO becomes overexpressed compared to its expression in normal tissues, making it a marker of immune system activation in the brain (29, 30). Multiple reports have validated TSPO as a marker of immune function, demonstrating a tight relationship between immunohistochemical measures of microglial activation and TSPO levels (31, 32). The effects of chronic substance use on TSPO expression in the brain have been examined in recent years using positron emission tomography (PET) alongside radiotracer ligands that bind to this mitochondrial transporter. Numerous radiotracers have been used in detecting TSPO distribution or levels in neuroinflammation, [^11^C]PK11195 being the earliest (33) and most widely known. High non-specific detection rate, short half-life, low brain uptake of [^11^C]PK11195, and/or low signal to noise ratio (SNR) were the main limitations for its reduced clinical utility over time, which led to the development of second generation radioligands with increased detection and SNR, including [^11^C]PBR28, [^11^C]DAA1106, [^11^C]DPA713, [^11^C]vinpocetine, [^11^C]DAC, [^18^F]PBR06, [^18^F]DPA-714, [^18^F]PBR111, and [^18^F]FEPPA. However, huge individual differences in TSPO binding potentials of these PET tracers is due to variation in a single nucleotide polymorphism in the TSPO gene (34). In humans the TSPO gene is located on chromosome 22q13.3. Of the four exons, single-nucleotide polymorphism (SNP; rs6971) in exon 4 of this gene leads to non-conservative substitution of alanine by threonine, which influences the ligand affinity of TSPO (34). This SNP results in three distinct TSPO binding statuses: high affinity (Ala/Ala), mixed affinity (Ala/Thr), and low-affinity (Thr/Thr) binders, necessitating time-intensive genetic testing to validate participant eligibility prior to PET scans (34). A third-generation tracer insensitive to the rs6971 polymorphism has been developed called [^11^C]ER176. Although this tracer exhibits adequate binding potential, enhanced specificity and SNR (35). Further details on different radiotracers, their design, and PET performance in probing neuroinflammation in both human and preclinical models has been detailed in some of the recent reviews (35–37).

Long-term cannabis users were shown to have increased TSPO levels in various brain regions. Specifically, a PET study on 24 long term cannabis users reported higher [^18^F]-FEPPA (a radiotracer for TSPO) total distribution volume (V_*T*_) in the dorsolateral prefrontal cortex (dlPFC), medial prefrontal cortex (mPFC), anterior cingulate cortex (ACC), temporal cortex, and cerebellum relative to controls. Moreover, greater TSPO levels in these brain areas were associated with stress and anxiety and higher circulating C-reactive protein (CRP) levels in cannabis users (38). This correlation between elevated TSPO and psychological alterations like chronic stress and anxiety in cannabis users aligned with several preclinical reports (39–41). Their results provide evidence for the exacerbation of neuroimmune activation as an effect of cannabis overuse, which mediates brain structure and function alterations. Further they observed a negative association of [^18^F]FEPPA V_*T*_ with life time cannabis use but not with past year cannabis use in dlPFC, mPFC, ACC, and temporal cortex (38). However, some of these effects may be substance-specific. Brody et al. used [^11^C]DAA-1106 to determine the impact of nicotine use in microglial activation, and they found reduced TSPO binding in the brain of smokers compared to non-smokers. Also, lower [^11^C]DAA1106 standard uptake value (SUV) was associated with more cigarettes smoked/day (42). Narendran et al. measured TSPO levels with [^11^C]PBR28 and found no significant differences in cortical and sub-cortical binding between individuals who misuse cocaine and healthy controls. Their study also demonstrated no significant association between [^11^C]PBR28 V_*T*_ in regions of interest and the duration of cocaine use in years (43).

Hillmer et al. used the TSPO ligand [^11^C]PBR28 in people with alcohol use disorder (AUD) and found significantly lower binding in the cerebellum, frontal cortex, hippocampus, and striatum compared with healthy controls (44). Their results also revealed a significant negative relationship of cerebellum, striatum, and hippocampal [^11^C]PBR28 V_*T*_ with reported drinks per day over the previous month and alcohol dependence severity, hinting at a connection between chronic alcohol use and microglial activation (44). Further, lower [^11^C]PBR28 V_*T*_ was reported in the hippocampal region of alcohol dependents compared to healthy control subjects (45). Using [^11^C]PBR28 and PET, we recently reported reduced binding only in AUD individuals with the medium-affinity TSPO polymorphism. Plasma cholesterol levels correlated negatively with whole brain [^11^C]PBR28 V_*T*_, mostly in AUD individuals. The five transmembrane helical structure of TSPO is also known to facilitate the transport of cholesterol across the outer mitochondrial membrane. Therefore, the reduced binding of the tracer in our study might account for increased plasma levels of cholesterol that competed with PBR ligand (46). In contrast, there were no differences *in vivo* binding of [^11^C]PBR28 but significant increases in the binding when performed *in vitro*, suggesting that an endogenous ligand with alcohol exposures interferes with the binding *in vivo*. Our data shows that the increased plasma levels of cholesterol in AUD might account for the reduced TSPO ligand binding in AUD, limiting the utility of this marker to monitor inflammatory responses in AUD (46).

In contrast, a recent study reported an upregulation in TSPO mRNA levels in the amygdala and FC from post-mortem tissues of AUD patients relative to healthy controls. The authors suggested that activation of immune responses and TSPO elevation as an effect of chronic alcohol intake is epigenetically regulated by histone deacetylases (HDACs), which regulate gene expression by removing acetyl groups from histones. HDACs are classified based on their distinct roles in immune function: Class I (HDAC 1, 2, 3, and 8) are involved in innate immunity and cytokine production, and Class II (HDAC 4, 5, 6, 7, 9, and 10) are involved in adaptive immunity. Interestingly, they found greater HDAC2 and HDAC6 mRNA levels in the amygdala tissue of AUD individuals compared to controls but not in NAc, hippocampal, or PFC tissues. The elevation of both TSPO and HDAC2 and HDAC mRNA levels in amygdala tissue perhaps indicates the unique sensitivity of the amygdala to epigenetic regulation and neuroinflammation in response to chronic alcohol exposure (47). The discrepancy across *in vivo* and *in vitro* studies on TSPO levels on chronic alcohol exposure unfolds various role played by TSPO including neuroinflammation, steroid hormone synthesis, various intracellular functions like apoptosis, oxidative stress, as well as its ability to regulate development of tolerance to alcohol (48).

### Interrelationships Between Inflammation, Cognitive Function, and Brain Metabolite Changes in Chronic Substance Users

Certain brain metabolite levels, such as N-acetyl aspartate (NAA) + N-acetyl-aspartyl-glutamate (NAAG) and NAA/Creatine (Cr), may serve as a surrogate for inflammation status. These appear to differ between acute and chronic alcohol users, indicating a dose-response relationship between alcohol intake and markers of microglial activation (49). For example, Bagga et al. reported an association between chronic alcohol use and lower NAA/Creatine (Cr) levels determined by *in vivo* proton magnetic resonance spectroscopy (MRS) in the visual cortex (50). Whereas, Haarman et al. reported a positive association between acute alcohol use and hippocampal NAA and NAAG levels (51). The levels of NAA and NAAG and total NAA in right inferior frontal cortex (IFC) were also associated with duration of metamphetamine use (52, 53). Perhaps the NAA + NAAG increase in substance users is caused by microglial activation attracting and activating other immune-competent cells, increasing NAA + NAAG concentrations. Moreover, microglia are involved in diverse roles, including tissue regeneration and neuronal support. It might be possible that some microglial cells actively facilitate neurogenesis while others induce apoptosis (54), which may explain the increased NAA + NAAG with substance use.

Numerous studies have reported lower NAA levels and a lower NAA/Cr ratio in methamphetamine users compared to healthy controls in frontal brain regions, in particular the anterior cingulate cortex (ACC) (55–59). Further, NAA/Cr ratio in the ACC was positively associated with attentional control (60) while it negatively correlated with duration of methamphetamine use (57) among methamphetamine users, and interestingly the ratio of these metabolites tended to recover, albeit slowly, with extended duration of abstinence (57, 61). However, acute methamphetamine abstinence was associated with lower NAA/Cr+PCr and NAA + NAAG/Cr + PCr levels in the frontal region (dorsolateral prefrontal cortex). This reduction in metabolite levels persisted through short-term methamphetamine abstinence which might be a consequence of decreased neuronal integrity or reduction in synthesis and degradation of cell membranes due to inflammatory processes initiated by methamphetamine abstinence (62).

Glutamate (Glu), another neurometabolite, is elevated in the thalamus of AUD individuals compared to healthy controls; greater Glu levels in the thalamus were further associated with the severity of alcohol drinking behavior. Elevated Glu levels have been associated with alcohol-induced neurotoxicity and neuroinflammation (63). Moreover, the same study reported a positive relationship between elevated Glu in the thalamus and dorsal ACC with impulsivity measures in chronic drinkers, thereby indicating a role of alcohol-induced neurotoxicity in exacerbating cognitive impairments (64). Further lower Glu+Gln levels in right insular region of metamphetamine users was negatively correlated with depressive symptoms and anxiety state (52). The same group further found that during early abstinence the Glu+Gln levels in the right inferior frontal cortex was inversely associated with occurrence of depressive symptoms (53). Methamphetamine-induced inflammation and neurodegeneration are likely behaviorally relevant, as peripheral markers of immune activation are associated with impaired cognitive functioning (65) (Supplementary Table 1).

### Association Between Inflammation and Brain Atrophy in Alcohol Use Disorders

Inflammation in chronic alcohol drinkers was associated with brain atrophy (66). Widespread structural atrophy appears to be a defining effect of chronic alcohol use (67). Ethanol mainly leads to white matter atrophy due to altered myelin synthesis (68). Neurodegeneration of hippocampal circuits also occurs (69). Although some effects are directly derived from ethanol and/or acetaldehyde, inflammation and oxidative damage may play a role (70, 71). Fibroblast growth factor (FGF)-23 (a hormone produced mainly by osteocytes in response to high serum phosphate levels) and Klotho (an obligate coreceptor of FGF-23) were hypothesized to be associated with brain atrophy in AUD (66). The secretion of FGF-23 by osteocytes is regulated differently in acute and chronic inflammation states (72–74). Some reports suggest that FGF-23 and Klotho increase in AUD. Given the pro-inflammatory effects of chronic alcohol use, especially with associated liver cirrhosis (75), it could be postulated that inflammation might play a role in these changes.

On the other hand, excessively high FGF-23 serum levels may be associated with impaired learning and memory (76), as observed in chronic kidney disease (77). The opposite is observed with Klotho (78). Contrarily, in the study by González-Reimers et al., AUD patients had lower levels of Klotho than controls. Klotho was directly related to TNF-α and inversely to TGF-β, but not CRP. These levels of Klotho were inversely associated with brain atrophy in AUD measured *via* computed tomography (CT), such that the levels were inversely related to several CT indices: bicaudate (minimum width of lateral ventricles/skull width at the same level), bifrontal (maximum width of frontal horns/skull width at the same level), Evans (maximum width of frontal horns/skull width at the level of the III ventricle) and a trend with cella index (width of the III ventricle/skull width at the same level).

Nevertheless, the levels of Klotho were notably higher among people with cirrhosis. Their results further demonstrated elevated FGF-23 in AUD, especially with cirrhosis, aligning with other reports. Notably, unlike Klotho, they did not observe a significant relationship between FGF-23 levels and brain atrophy in AUD; FGF-23 levels were not inversely related to the bifrontal index (66) (Supplementary Table 1).

### Inflammatory Response in Severe and Abstinent Individuals With a Substance Use Disorder

Though PET TSPO ligands might be confounded in studies of AUD due to competition with cholesterol, some have interpreted the reduction in [^11^C]-PBR28 binding in the hippocampus and striatum as indicative of impaired microglia activation. This was supported by an inverse association between TSPO binding in these brain regions and AUD severity and reported drinks per day over the previous month (44). Moreover, microglial expression and activity may depend on the duration and severity of AUD, such that the activated microglial levels diminish with a more significant duration of alcohol misuse (44). It is documented that the neuro-immune system activation in chronic alcohol drinkers impacts their neurobiology throughout the three established stages of the “addiction cycle”: binge/intoxication, withdrawal/negative affect, and preoccupation/craving (79) [detailed in review by Crews et al. (80)]. Furthermore, individuals with AUD struggle with craving during abstinence, and some previous reports showed a positive association between craving and TNF-α and IL-6 levels in peripheral blood during alcohol withdrawal (81). A recent study in AUD individuals at 1 month of abstinence investigated the link between inflammatory biomarker expression and craving in the AUD population (82). They observed a significant increase in the plasma levels of IL-6, CRP, and TNF-α in AUD patients after 1 month of abstinence. Based on their results, brain-derived neurotrophic factor (BDNF) and inflammatory cytokines were proposed as potential biomarkers for craving severity in post-abstinent AUD (82).

Methamphetamine is also known to alter immune function significantly. However, post-abstinence, microglial levels may decline as an effect of treatment. One recent study found using the TSPO PET ligand [^11^C]-DAA1106 that binding was not different in the whole striatum of recently abstinent (<6 months) methamphetamine-dependents compared to controls (83). However, a study that used the TSPO ligand [11C](R)-PK11195 reported a negative correlation between binding in the midbrain, striatum, and thalamus and duration of methamphetamine abstinence (84). Thus, microglial activation may increase in the brain during earlier abstinence, consistent with the increased cerebral glucose metabolism during the first month of abstinence (85), but then wanes as the brain heals during long-term abstinence (Supplementary Table 1).

### Microglial Activation in Mood Disorders

The development of comorbid conditions like bipolar disorders (BD) and in major depressive disorders (MDD) individuals with substance use disorders is strongly related to central and peripheral inflammation in these individuals (5, 86, 87). It is also well-documented that increased inflammatory marker levels like IL-3455 greatly influence the pathophysiology of depressive and mood episodes in MDD and BD patients (88), IL-6 (89, 90), IL-10 (89), and CRP (90), neuronally derived exosome marker CD81 (88), also TSPO in brain regions like PFC, ACC, and insula (91).

#### Bipolar Disorder

TSPO reportedly forms a complex with voltage-dependent anion channel (VDAC), which increases reactive oxygen species levels in mitochondria and inhibits mitophagy, leading to a pronounced accumulation of damaged mitochondria and an increase in NLRP3-dependent inflammation in BD (92). Du et al. found that at-risk individuals with anxiety disorders who have markedly higher IL-6, TNF-α, and CRP at baseline were more likely than others to develop BD during an 18-month follow-up period (93). In addition, compared to controls, IL-8 levels were higher in cerebrospinal fluid from BD patients relative to controls (94). No microglial activation was observed in post-mortem brain tissues of BD patients (95–98). Moreover, microglia activation was not abnormal in most brain regions of BD patients, except for the hippocampus on PET examination with [^11^C]-(R)PK11195 (99).

In psychosis, on the other hand, findings from several studies suggest the BBB is damaged (100). The increased permeability of the BBB allows inflammatory molecules and immune cells to enter. These act as disease modifiers in genetically predisposed individuals by interacting with neurotransmitter systems that might contribute to the disease. Lymphocytes located outside blood vessels and in the brain parenchyma are distinct signs of neuroinflammation or vascular disorders like multiple sclerosis, viral CNS infections, and ischemic stroke (101). In particular, classical immune cells like T and B lymphocytes do not enter the healthy brain tissue in more significant numbers; instead, these cells stay within the blood vessels. T cells and, to some extent, B cells were found to be increased in brain regions like hippocampus/parahippocampus, as well as in the thalamus, temporal and frontal cortex, cingulate gyrus, and white matter in about one-third of BD patients (102). In a subsequent study by Schlaaff et al., CD3+ T and CD20+ B cellular densities in whole brain sections were elevated in 7 out of 20 BD patients (103) (Supplementary Table 2).

#### Major Depressive Disorder

Increased CRP levels are associated with increased anxiety and depression risk. Moreover, a correlation between high CRP levels and threat-related amygdala activity (a neural biomarker of depression and anxiety risk) as assessed with functional MRI (fMRI) during an emotional face-matching task, specifically in male undergraduate students was reported. These results add to our knowledge on the critical role of threat-related amygdala activity in heightening chronic inflammation and thereby increasing the risk for mood or anxiety disorders (104). The prospective Whitehall II cohort study reported that over 12 years, participants with a high IL-6 level at baseline had a higher risk of depressive and anxiety disorders at follow-up than those with a low IL-6 level at baseline (105). Higher IL-4 and IL-6 levels were seen in MDD compared to healthy control subjects (106). In addition, compared to controls, IL-8 levels were elevated in post-mortem whole brain tissue from MDD patients (25). Although previous studies have hypothesized a role for microglia in the pathogenesis of MDD (107), some of the most recent studies on post-mortem tissues from MDD patients reported contradictory findings suggesting a role of microglia in homeostatic functions, brain circuit development, and maintenance but no signs of immune activation in brain regions (frontal lobe, temporal lobe, thalamus, and subventricular zone) of depressive disorder patients (108, 109). These findings emphasize how neuronal damage and microglial dysfunction may be critical components underlying the pathophysiology of MDD and BDs.

### Interrelationships Between Inflammation, Cognitive Function, and Brain Volumetric and Metabolic Changes in Mood Disorders

#### Bipolar Disorder

Early stage BD is associated with elevated concentrations of pro-inflammatory cytokines, such as IL-10 (110). PET-MRI research showed a decrease in hippocampal volume of BD patients (51), but no significant differences in [^11^C](R)-PK11195 binding of the left hippocampus of BD patients compared to healthy controls. However, critically they observed lower NAA + NAAG concentrations in the left hippocampus of BD patients relative to controls. They also found an association between microglial activation and left hippocampus NAA + NAAG and depression severity in BD patients. The authors argued that perhaps an NAA + NAAG increase is caused by microglial activation attracting and activating more immune-competent cells, thereby directly increasing the NAA + NAAG concentrations. This is consistent with the neuroinflammation theory postulated by Stertz et al. (111), which proposes that following the first acute mood episode, neuronal injury leads to the release of damage-associated molecules that activate microglia. The activated microglia, in turn, release both pro-inflammatory cytokines and neurotrophic factors. These factors then modify the synaptic environment by a mechanism known as synaptic pruning as an adaptation to survive the adversity caused by the acute disease episode. Then, after several episodes, the increased production of pro-inflammatory cytokines, exceeding average capacity, maintains the microglia in a constantly activated state (111).

The neurotrophin BDNF has emerged as a crucial mediator of neuronal plasticity since it is abundant in brain regions particularly relevant for plasticity (neurogenesis) and shows a remarkable activity-dependent regulation of expression and secretion (112), suggesting that it might bridge experience with enduring changes in neuronal function. Furthermore, several lines of evidence indicate that inflammatory markers reduce BDNF levels (113–115), thereby contributing to the development of psychiatric diseases. Early stage BD is characterized by neurocognitive dysfunction, particularly deficits in psychomotor speed, attention, working memory, cognitive flexibility, and executive functioning, including verbal learning, attentional switching, and verbal fluency (116). Various studies have explored an interrelationship between cytokine levels in different brain regions and cognitive alteration in BD patients. BD patients showed impaired executive functioning on a frontal assessment battery (FAB), especially in interference, inhibitory control, and increased BDNF plasma levels compared to controls. However, no correlation between BDNF levels and executive impairment was noticed in BD patients (117). A recent study reported an association between greater BDNF levels in euthymic and manic BD patients with disrupted executive function in the Wisconsin Card Sorting Test and verbal memory in the California Verbal Learning Test (118).

#### Major Depressive Disorder

During the early stages of MDD, including acute and remitted phases, higher TNF-α and IL-6 were reported compared with controls [review by Himmerich et al. (119)]. Reports on cognitive impairment in MDD patients is heterogenous due to reasons like differences in patient population (like symptom severity, depressive subtype, comorbid conditions), as well as variation in cognitive tests used for assessment across studies (120). The cognitive dysfunction in MDD patients has been attributed to increased expression of various pro-inflammatory cytokines, dysfunctional mitochondrial functioning, hyperactivity of the hypothalamic-pituitary-adrenal gland (HPA) axis, as well as decreased neurotrophic factors (121). It has been noted that individuals suffering from depressive disorders reveal volumetric changes in whole-brain gray matter and specific brain regions, including the orbitofrontal cortex, lingual gyrus, inferior frontal cortex, middle frontal cortex, planum polare, and hippocampus, which were inversely correlated with cytokine levels in these patients [detailed in review by Han et al. (122)]. A meta-analysis documented that first-episode MDD patients have lower brain volume than controls (123). Moreover, elevated TSPO levels were seen in several brain regions (white matter, gray matter, frontal cortex, temporal cortex, and hippocampus) of MDD patients using [^18^F]-FEPPA PET. Significantly, the increased TSPO V_*T*_ in the frontal cortex was associated with lower scores on the Repeatable Battery for the Assessment of Neuropsychological Status attention domain (124).

Together, these findings suggest an impact of pro-inflammatory processes on brain function and cognitive impairment in mood disorders. One possible biological mechanism could be the interaction between altered cytokine levels and HPA stress axis function, which may alter synaptic plasticity and eventually lead to cognitive deficits in these individuals (125). Hyperactivity in the HPA axis, exhibited as elevated cortisol levels, is one of the most consistent findings in BD and MDD (126–129). Further, one study found a negative correlation between whole blood mRNA expression of IL-1β and cortisol reactivity, indicating a link between HPA and immune dysregulation in depression (130). In a different study depressed patients with and without lifetime hypomanic episodes were compared, and their results demonstrated no association between cortisol indicators or inflammatory markers and lifetime hypomanic episode. However, their results revealed that the association between diurnal cortisol slope and a lifetime hypomanic episode in depressed males depended on CRP level. Depressed men with higher diurnal cortisol slope and CRP levels had high probability for presenting with a hypomanic episode. The results obtained further highlighted the importance of simultaneous assessment of HPA axis function and inflammation to differentiate bipolar from unipolar disorders (131). Of note, one of the studies demonstrated an inverse relationship between diurnal salivary cortisol levels and hippocampal Glu and NAA concentrations in bipolar patients. This finding reaffirms the relationship of HPA stress axis and disturbed neuroplasticity in BD patients (132).

The activity of the HPA axis varies among subtypes of depressive disorders. Hyperactivity of the HPA axis, as demonstrated by higher ACTH and cortisol levels, was noted in melancholic depressive disorder patients compared to non-melancholic patients. However, significantly lower cytokine levels of IL-1h and IL-1RA were seen in melancholic patients, while the levels increased in non-melancholic patients before remission (133). Frodl et al. found that MDD patients with less expression of the cortisol markers glucocorticoid-inducible Leucine Zipper (GILZ) or glucocorticoid-inducible kinase-1 (SGK-1) had smaller hippocampal volumes. Critically, they observed a strong positive association of GILZ or SGK-1 mRNA expression and hippocampal volume and a negative correlation of IL-6 levels with hippocampal volume in MDD patients. Overall, their findings point to interrelationships between cortisol, inflammation, and hippocampal volume in the pathophysiology of MDD (134).

Further, some studies have explored the relationship between pro-inflammatory cytokines, gray matter volume, and cognitive changes in first-episode BD and MDD patients. Chen and colleagues conducted their survey on adolescent and adult BD/MDD patients. Their results revealed higher tumor necrosis factor-α receptor 1 (TNF-αR1) levels, poor Wisconsin Card Sorting Task executive function, and significant gray matter volume reduction in the left middle frontal cortex (MFC) first-episode BD patients compared to both first-order MDD and healthy controls. Meanwhile, patients with first-episode MDD exhibited lower gray matter volume in the right occipital fusiform cortex and left postcentral cortex than controls. As hypothesized, they also observed a positive association between left MFC volume and both TNF-αR1 levels and executive function in patients with first-episode MDD. The results highlight the complex patterns of how brain structure and systemic inflammation are associated with first-episode affective disorders (135) (Supplementary Table 2).

### Inflammatory Response in Severe Mood Disorders

Neuropathological progression associated with severity of MDD and BD has been related to inflammatory processes like loss of astroglia and consequently reduced glutamate uptake, loss of somatostatin positive interneurons, decreased neurogenesis, the persistence of greater monoamine oxidase A-concentrations, hippocampal volume loss, and chronic microglial activation (91, 136–140). Neuroinflammatory markers like TNFR1/CD81 correlated positively with severe “behavioral” symptoms, including agitation in MDD patients (88). While, in BD patients IL-8 levels varied with the symptom severity such that the most severe BD-I patients showed higher IL-8 levels than the BD-II and other specified bipolar patients (141). A systematic review conducted on BD patients revealed lower BDNF levels during manic and depressive episodes compared to control subjects. The findings were heterogenous between euthymic BD and controls (142). However, van den Ameele et al. reported no significant differences in neurotrophic factors like BDNF, VEGF, and sFlt-1 levels in manic and depressive BD patients compared to healthy controls. Moreover they did not see a significant correlation between these neurotrophic markers and mood symptom severity of BD patients (143). In contrast, Köse Çinar et al. observed lower BDNF mRNA expression in severe manic BD patients than healthy controls. Further, increased BDNF expression was seen in remission compared to severely manic BD patients, indicating the possible value of this biomarker in tracking disease progression (144). The severity of manic and depressive symptoms was also positively correlated with NLRP3 inflammasome components and TSPO/VDAC expression levels, respectively (92).

A small cohort [^11^C]-PBR28-PET study on acute episode MDD patients showed lower TSPO levels (lower [^11^C]-PBR28 V_*T*_) in frontal, temporal, parietal, and occipital cortex, caudate, putamen, thalamus, cerebellum, and white matter (centrum semiovale) (145). In contrast, a PET [^18^F]-FEPPA study revealed a significant elevation in TSPO V_*T*_ levels in the frontal cortex, temporal cortex, and hippocampal regions in MDD patients before treatment. However, a considerable reduction in TSPO V_*T*_ was seen post cognitive-behavioral therapy (CBT); that in the hippocampus correlated with the improvement in their depressive symptoms consistent with reduced pro-inflammatory activity following treatment (146). Setiawan et al. measured if [^18^8F]-FEPPA-PET binding was related to the duration of untreated depressive disorder (short course: for 9 years or less; long period: for 10 years or longer). Their found 29–33% higher TSPO V_*T*_ in the PFC, ACC, and insula of long duration than short-duration untreated MDD patients.

Moreover, TSPO levels were 39% higher in these brain regions of MDD patients with a longer untreated disease duration than healthy participants. Therefore, their finding suggested increased microglial activation with chronicity in depressive disorders (147). This finding was confirmed by a later [^11^C](R)-PK11195-PET study, wherein they investigated TSPO levels in a cohort of medication-free patients in a major depressive episode of at least moderate severity that was compared with matched healthy subjects. They also observed greater TSPO levels, particularly in ACC of the MDD cohort relative to controls, and greater TSPO in ACC and insula of MDD patients experiencing suicidal thoughts compared to patients without suicidal thoughts.

These findings indicate how inflammatory function may play a role in suicidal thinking in MDD, a predictor of whether the illness will be treatment-resistant (138). In line with this, higher plasma concentrations of BDNF and IL-1β were observed in treatment-resistant depressive patients relative to controls (148). Two potential mechanisms for inflammation-induced persistent depression are (i) TNF-α-associated reduction in serotonin and BDNF levels (149), and (ii) chronically elevated amount of glutamate in the synapses and subsequent neural toxicity (150, 151). To assess if increased TNF-α may impact brain function, as well as glutamatergic neurotransmission in treatment-resistant MDD patients, an ^18^F-FDG-PET study was conducted in treatment-resistant and non-resistant MDD groups. Higher serum concentrations of TNF-αR1 in treatment-resistant patients were observed compared to those who responded to treatment and showed a significant positive association between TNF-αR1 levels and glutamatergic neurotransmission in caudate and ACC in the treatment-resistant group. Together, these data provide compelling evidence that neuroinflammation is a critical target for novel therapies addressing treatment-resistant MDD (152) (Supplementary Table 2).

### Inflammatory Response in Comorbid Substance Use Disorder and Mood Disorders

**FIGURE 1:**
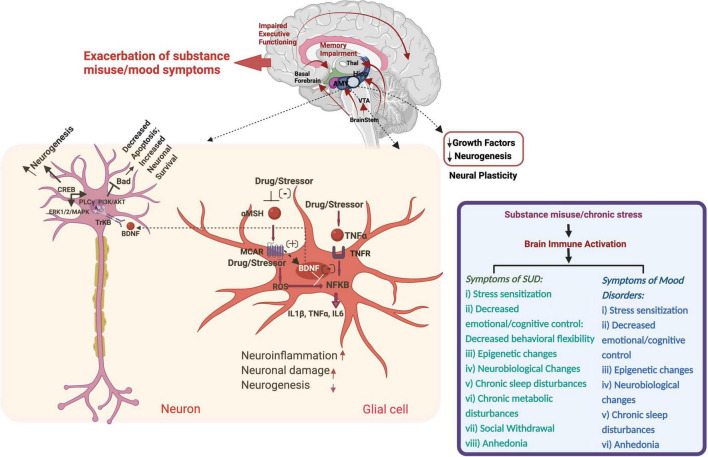
Schematic of substance- and stress-induced neuroinflammation and pathophysiological changes in substance use and mood disorders through immune system modulation. Thal, Thalamus; Hipp, Hippocampus; AMY, Amygdala; VTA, Ventral Tegmental Area; CREB, cAMP Response Element Binding Protein; Bad, BCL2 Associated Agonist of Cell Death; PLC, Phospholipase C; PI3K, Phosphoinositide 3-kinases; AKT, Serine/Threonine Kinase; ERK, Serine/Threonine Protein Kinase; TrKB, Tropomyosin Receptor Kinase B; BDNF, Brain-Derived Neurotrophic Factor; MSH, Melanocyte Stimulating Hormone; TNF, Tumor Necrosis Factor; ROS, Reactive Oxygen Species; IL, Interleukin.

The relationship between substance and mood disorders is getting much attention. During the addiction development phase, downregulation of the reward system is associated with recruitment of stressors which contribute to negative emotional states, with a consequent increase in craving for repeated substance intake (153). Moreover, stress exacerbates the risk of drug abuse and relapse (154), as well as depressive episodes (155) (Figure 1). A study assessing the reactivity of HPA axis between non-substance-abusers, polysubstance-abusers without depressive symptoms, and with both substance abuse and depressive symptoms revealed blunted adrenocorticotropic hormone and cortisol responses to ovine corticotrophin-releasing hormone (CRH) administration in polysubstance abusing subjects with no past or current diagnosis of depressive disorder (156). This finding hints at an overlapping role of CRH and HPA axis activation in both mood and substance use disorders (156). A population-based study demonstrated lower IL-6 levels in men and lower TNF-α levels in both genders with a lifetime BD diagnosis; lower levels of IL-6 and TNF-α were also reported in the unspecified subtypes of lifetime MDD population while increased hsCRP levels were noted in men with lifetime SUD diagnosis (157). Further several correlations between lifetime clinical characteristics and pro-inflammatory markers were noted in adolescents and young adults with BD. For example, associations were noted between longer illness duration, IL-6 and hsCRP; suicide attempts and self-injurious behavior with TNF-α; family history of suicide attempts or completion and SUD with hsCRP; and family history of SUD with TNF-α, and IL-6 (158). Also there are many papers stating a “cross-sensitization” between stress and substance of abuse (159–162). An important clinical implication of the cross sensitization theory is the challenge associated with the management of stressful and emotional distress during abstinence, which often leads to relapse (163).

Some studies have reported collective roles of oxidative stress and inflammation in mood and substance use disorders. Smokers with depressive symptoms compared to non-depressed never smokers showed both higher (nitrosative stress, fibrinogen, hs-CRP, advanced oxidation protein product, erythrocytes sedimentation rate, IL-6, TNF) and lower (total radical antioxidant potential) levels of inflammation and stress markers (164, 165). Anxious smokers revealed significant polymorphisms in glutathione-S-transferases M1 (GSTM1) and T1 (GSTT1) genes, with no difference in unipolar and bipolar smokers. They observed a greater likeliness mood disorders and suicidal attempts in anxious smokers than non-anxious smokers (166). Further the same group reported an association between comorbid mood and tobacco use disorder and Tin2.10/10, GSTM1, and GSTT1 genotypes and lowered paraoxonase 1 activity (167, 168). Additionally, there are increased odds of BD in smokers who are homozygous for the PON1 QQ genotype and the risk for MDD was predicted by plasma PON1 expression, as well as interactions between genotype and smoking (169). There are also some reports on inflammatory markers in comorbid MDD and AUD subjects. The results for BDNF levels were heterogenous, where some found no significant difference in depressive patients with and without alcohol dependence (170, 171), but others report lower levels in AUD patients with depressive states (172). AUD patients with comorbid MDD presented with higher insulin-like growth factor, IL-6, TNF-α, IFN-γ (173) and tryptophan concentrations (174) but lower nerve-growth factor and IL-10 levels (171, 173). Genetic studies have noted significantly higher frequency of the A allele of rs6265 (Val66Met) in AUD with depression compared to those without depression (175). However, others reported no association between BDNF Val66Met polymorphism and depression in alcohol dependents (176, 177).

## Limitations

This review has some limitations to note. The initial plan (pre-registered on Prospero at https://www.crd.york.ac.uk/prospero/display_record.php?RecordID=238128) was to perform a systematic review and meta-analysis, asking the question: which neuroinflammation biomarkers exhibit different rates in people with SUDs and/or people with mood disorders, relative to their healthy control counterparts? However, after the initial search was carried out it became clear that there were too many across-study differences in methodology, specific biomarkers assessed, and brain regions measured to provide meaningful comparisons. Thus, while the original search strategy was structured, we ultimately had to abandon formal statistical analysis of the findings presented here. As more studies are published with overlapping methodology, it will be valuable for future studies to perform meta-analysis, and also to assess risk of bias and confidence in the evidence in a more formal manner.

## Conclusion

In summary, the literature cited in this review provides evidence for the role of inflammatory factors in the etiology of SUD, depression, and mood disorders. Moreover, the study highlights converging evidence for a bidirectional relationship between activation of inflammatory response and the onset and persistence of SUD and comorbid conditions like depression and mood disorders.

Neuroinflammatory processes may differ across the different forms of SUD. PET investigations in various SUDs have revealed variable levels of neuroinflammation relative to healthy control populations: TSPO appears elevated in cannabis use disorder, not significantly different in cocaine misusers, and lower in alcohol and nicotine use disorders. However, TSPO mRNA levels were elevated in post-mortem amygdala tissue of AUD individuals and rodent models of AUD. PET studies further suggest that TSPO levels are inversely related to the severity of AUD, specifically in the hippocampus and striatum. Data also imply an association between an activated inflammatory response and treatment resistance in a subset of patients. High concentrations of inflammatory cytokines like TNF-α and IL6 may serve as biomarkers for determining the extent of craving in AUD individuals in early abstinence. However, long-term abstinence appears to lead to decreased microglial activation.

Reports consistently suggest that increased IL-6, TNF-α, and CRP levels are risk factors for mood and depressive disorders. fMRI studies revealed a positive relationship between CRP levels and threat-related amygdala activity in mood disorder patients. Further, there appears to be a relationship between cytokine levels in different brain regions and cognitive alterations (especially deficits in executive function) in both bipolar and MDD patients. One possible biological explanation is the interaction of pro-inflammatory cytokines and hyperactivity in the HPA axis, which influence neuronal plasticity and negatively impact mood symptoms and cognition. The levels of neurotrophic factors and inflammatory markers reportedly vary with the severity of mood and depressive symptoms. BDNF is the most studied factor, with lower levels reported in severe BD patients relative to patients in remission and healthy controls. Lower TSPO levels were seen in several brain regions, including frontoparietal and dorsal striatal brain regions during the acute stages of MDD, relative to controls. However, MDD treatment reduced TSPO levels in the hippocampus due to decreased depressive symptoms. Furthermore, higher TSPO levels are associated with more significant MDD disease duration. Studies have also shown that inflammatory markers may discriminate between treatment-resistant and non-resistant mood disorder patients, with the augmented levels TNF-α, IL-1β, and BDNF as markers of poorer antidepressant response.

Specific inflammatory markers such as TNF-α, IL-1β, CRP, and TSPO may be commonly involved in the pathogenesis of both SUD and mood disorders. On deeper inspection of the studies included in the present review, we observed overlap in the brain regions affected that included PFC, ACC, amygdala, hippocampus, thalamus, occipital cortex, temporal cortex, and cerebellum where abnormal levels of these inflammatory markers were seen SUD and mood disorder patients. The levels of NAA + NAAG in the left hippocampal region of substance (alcohol) and mood (bipolar) disorder patients yielded opposite results: in AUD, it was higher than controls, whereas, in bipolar disorder, levels were lower than controls.

However, we see inconsistencies across literature reports which may be attributed to the influence of multiple factors, including (i) the varying degrees of inflammatory vs. anti-inflammatory properties of each inflammatory marker, (ii) the variability in the methodology adopted to measure the inflammatory response, highlighting the need for a more standardized approach to measuring peripheral and central inflammation, (iii) finally, it is essential to consider the heterogeneity and severity of substance use (type of drug, dose, and frequency), depressive and bipolar disorder individuals who greatly influence their inflammatory activation status and are crucial to identifying successful novel therapies (Table 1).

**TABLE 1:** Unresolved questions deserving future study.

	Unresolved question	Possible causes for discrepancies/Lack of knowledge
1.	Are there consistent sex differences in inflammatory changes in SUD/mood disorders?	Lack of sex hormone measurement; small sample sizes; acute vs. chronic effects of drugs on inflammation may differentially affect males and females.
2.	How can cannabis help alleviate some symptoms in inflammatory disorders, but chronic use leading to a cannabis use disorder is associated with heightened inflammation?	Lack of longitudinal studies on how chronic cannabis use is associated with changes in inflammatory markers.
3.	What is the impact of drug-induced overdose on neuroinflammation?	No studies have comprehensively assessed this, nor determined whether factors such as hospitalization or overdose reversal with naloxone affect inflammatory outcomes.
4.	Does chronic alcohol use lead to an increase or decrease in the inflammatory marker TSPO?	*In vivo* and *in vitro* studies have shown different results, which may be due to limitations of the methodology (e.g., binding of the PET tracers may be sensitive to individual differences in TSPO genetic polymorphism variation, and competition of cholesterol with tracer binding).
5.	Why do metabolite levels differ in substance and mood disorder?	Mood disorders occur comorbidly with substance use but the levels of NAA + NAAG reported were higher in AUD while it was lower in bipolar disorder conditions when compared to controls. This might be due to methodological differences in MR spectrum processing across studies.

## Future Directions

Future studies need to focus on providing a comprehensive review on sex-specific differences in inflammatory changes in substance use and mood disorders, as considerable biological differences have been documented between males and females in terms of both acute and long-term effects caused by drugs. This has been partly attributed to differences in sex-hormone. Females tend to exhibit enhanced stress-related responsivity and show a more significant pro-inflammatory response marked by higher levels of TNF and IL-6 following acute administration of the drug compared to males (178). Also, IL-6 levels reportedly are elevated in females with depressive symptoms compared to healthy females and depressed males (179, 180). In contrast, increased severity scores have been linked to higher TNF in depressed males but lower TNF and IL-6 in female counterparts (181).

Further research should also be dedicated to highlighting the impact of drug-induced inflammatory changes associated with comorbid conditions in these individuals. Moreover, drugs like cannabis (commonly known as marijuana) are prescribed as complementary and alternative medicine to treat several chronic inflammatory diseases. However, the exact mechanism of its therapeutic role in these inflammatory conditions is still unclear, which warrants detailed investigation. It is also not known at what point chronic cannabis use becomes pro-inflammatory, since individuals with a cannabis use disorder show elevated inflammatory markers compared to controls. Yet another underexplored field of research is the effects of a drug overdose in neuroinflammation. The hypoxia associated with an overdose suggests that inflammation is a possible outcome, but the magnitude of the effects is unknown, and how much effects are moderated by the severity of the overdose (did it require hospitalization?) and interventions (was the person revived with naloxone, in the case of opioid overdoses?). The psychoneuroimmunological impact of SUD and comorbid mood disorders highlight the importance of targeted immunotherapies to treat these disorders. The studies summarized in the present review suggest the importance of developing pharmacotherapies to regulate and reduce inflammation and oxidative stress in individuals suffering from substance and mood disorders.

Yet another crucial future prospect would be a comprehensive review focused at specific neuroimaging studies in understanding neuroinflammatory mechanisms and associated cognitive, structural, as well as brain metabolite changes in autoimmune disorders like autoimmune encephalitis (AE) and autoimmune psychosis (AP) which are associated with mood problems. Inflammation-associated damages in limbic structures including hippocampus, cingulate cortex, temporal lobes, frontal basal regions in these patients causes the presentation of cognitive, mood and behavioral changes (182). Several reviews have summarized that cognitive deficits including persistent memory impairment in these patients are contributed by inflammation associated hippocampal atrophy (183, 184). Moreover, the clinical utility of PET imaging has been demonstrated due to its sensitivity in tracking metabolic changes in otherwise normal appearing brain structures of these patients (185–187). These reports call for a detailed review of studies highlighting the clinical outcome of neuroimaging studies conducted so far, highlighting how neuroinflammation-associated cognitive and metabolic changes in autoimmune disorders associates with psychotic symptoms and has therapeutic implications.

## Author Contributions

PM, NV, and PJ conceived the topic, planned the review, and co-wrote the manuscript. KA wrote the first draft of the manuscript. KA, MC, NN, EB, KM, ED, and AJ performed the literature search, selected final articles for inclusion in the review, and edited the manuscript. All authors contributed to the article and approved the submitted version.

## Conflict of Interest

The authors declare that the research was conducted in the absence of any commercial or financial relationships that could be construed as a potential conflict of interest.

## Publisher’s Note

All claims expressed in this article are solely those of the authors and do not necessarily represent those of their affiliated organizations, or those of the publisher, the editors and the reviewers. Any product that may be evaluated in this article, or claim that may be made by its manufacturer, is not guaranteed or endorsed by the publisher.
