# Supplementary material for: Inflammatory Markers in Substance Use and Mood Disorders: A Neuroimaging Perspective
Source: Front Psychiatry. 2022 Apr 26;13:863734. doi: 10.3389/fpsyt.2022.863734 (PMC9086785; doi:10.3389/fpsyt.2022.863734)
Supplement: Supplementary file 1 [file Table_1.docx]

**SUPPLEMENTARY TABLE 1** | Summary of Studies on Inflammatory Changes in Substance Use Disorder

| **S/No.** | **Author**  **(year)** | **Number of participants**  **(age; gender)** | **Methodology** | **Result** | **Interpretation** |
| --- | --- | --- | --- | --- | --- |
| 1. | De Carvalho et al. 2021(125) | Post-mortem tissues [PFC, NAc, hippocampus, and AMY] from:    AUD males  (n = 11, 50.6 ± 6.1 yrs)  HC males  (n = 13, 49.4 ± 12.4 yrs) | *qPCR* | 1. TSPO mRNA levels in AMY and PFC and HDAC2 and HDAC6 mRNA levels in AMY were upregulated in AUD compared to HC. 2. In AMY, the levels of TSPO mRNA correlated positively with HDAC2 and HDAC6 mRNA levels, suggesting the possibility of TSPO regulation by HDAC2 and HDAC6 in this brain region. 3. There are no group differences for TSPO, HDAC2, and HDAC6 in NAc and HPP. | The levels of TSPO mRNA in AMY and PFC in post-mortem brains from AUD consistent with neuroinflammation implicate epigenetic regulation of TSPO by HDAC2 and HDAC6. |
| 2. | Xu et al. 2020(82) | AUD  (n = 83, 39.16 ± 9.1 yrs)  HC  (n = 61, 42.39 ± 11.4 yrs) | *ELISA* – to measure the concentrations of nesfatin- 1, leptin, cortisol, BDNF; IL-6, CRP, and TNF-α | Average IL-6, CRP, and TNF-α levels in patients with AUD were significantly increased, while the BDNF levels were significantly decreased. | Inflammatory factors may play an essential role in AUD. |
| 3. | Huckans et al. 2015(87) | Active Methamphetamine Users (n = 17, 41.2 ± 10.3 yrs, 19% females)  Remission Methamphetamine Users (n = 36, 36.8 ± 9.5 yrs, 22% females)  HC  (n = 31, 397.6 ± 13.8 yrs, 32% females) | *ELISA* | 1. Methamphetamine dependents evidenced greater anxiety and depression during active use and remission, and more attention, memory, and executive problems during remission but not active dependence. 2. Ten immune factors (putatively associated with cytokine–cytokine receptor interactions) were associated with anxiety, depression, and memory problems in these patients. | Altered expression of a network of immune factors contributes to neuropsychiatric symptom severity. |
| 4. | London et al. 2020(83) | Methamphetamine-dependent participants abstinent for <6 months  (n = 11, 33.8 ± 7.5 yrs, 41.7% females)  HC  (n = 12, 38.3 ± 9.3 yrs, 18.2% females) | *PET scan* with [^11^C] -DAA1106 | SUVs for [^11^C]-DAA1106 were more significant in all brain regions of methamphetamine-dependent participants than controls. | Elevated binding in longer-abstinent methamphetamine users may reflect methodological differences or limitations of TSPO binding as an index of neuroinflammation.  Gliosis increases over time during the first six months of abstinence. |
| 5. | Wiers et al. 2020(64) | Recently detoxified AUD  (n = 15, 47.1 ± 9.1 yrs, 27% females)  HC  (n = 14, 48.0 ± 11.4 yrs, 28% females) | *DTI*  *In vivo ^1^H MRS* | 1. AUD patients revealed elevated Glu levels and lower FA values in thalamus but not in the vACC. 2. In AUD patients elevated thalamic Glu levels and FA were associated with drinking severity (drinks/week).   Increased impulsivity in AUD compared to HC showed an association with Glu levels in thalamus and vACC. | 1. This elevation in thalamic Glu levels and the parallel reduction in FA in AUD - which also correlated with drinking severity, could provide neurotoxicity from neuroinflammation.   Association of Glu with impulsivity suggests neurotoxic effects of chronic alcohol exposure in the thalamus and dACC as contributors of impulsivity. |
| 6. | Tang et al. 2019(52) | Methamphetamine users who agreed to remain abstinent for 2 weeks  (n = 45, 33.0 ± 9.3 yrs, 53.3% females)  HC  (n = 45, 32.9 ± 8.6 yrs, 51.1% females) | *MRSI* | 1. NAA + NAAG was elevated while, Glx was lower in the left insula and in the right inferior frontal cortex of methamphetamine users compared to HC. 2. NAA + NAAG was positively correlated with years of heavy methamphetamine exposure. 3. Glx negatively correlated with depression severity and anxiety symptoms in methamphetamine users. | 1. Increased NAA + NAAG levels could be explained by transient increased inflammatory response in early abstinence. 2. The negative correlation between Glx and severity and anxiety symptoms pertains to the possible relationship of glutamatergic systems with mood symptoms during early abstinence from methamphetamine. |
| 7. | Gonzalez-Reimers et al. 2018(66) | Alcoholics  (n = 131, 57.60 ± 11.11 yrs, 5.3% females)  HC  (n = 41, 54.49 ± 7.36 yrs, 14.6% females) | *Brain CT examination*  Serum MDA levels were measured as *TBARS.* | 1. Marked brain atrophy was observed among alcoholics when compared with HC. 2. Higher FGF-23 and lower Klotho values in alcoholics. 3. Among cirrhotics, Klotho values were higher. 4. Klotho was inversely related to brain atrophy [for instance, ventricular index, especially in cirrhotics. 5. Klotho was also directly related to TNF-α and inversely to TGF-β, but not to CRP or malondialdehyde levels.   FGF-23 was also higher among cirrhotics but showed no association with CT indices. | Increased soluble α Klotho levels among alcoholic cirrhotics compared with non-cirrhotic alcoholics is related to liver function impairment. Brain atrophy is inversely associated with α Klotho levels in alcoholics, especially among cirrhotics. |
| 8. | Burger et al. 2018(62) | Metamphetamine Dependent Acute Abstinence  (n = 31, 28.1 ± 5.3 yrs)  Metamphetamine Dependent Short Term Abstinence  (n = 31, 27.6 ± 5.1 yrs)  HC  (n = 22, 28.6 ± 10.2 yrs) | *In vivo ^1^H MRS: frontal region* | 1. Lower levels of NAA/Cr + PCr and NAA + NAAG/Cr + PCr was seen in both acute and short-term metamphetamine abstinents. 2. Lower concentrations of GPC + PCh/Cr + PCr were observed in the left frontal white matter of acute and short-term metamphetamine abstinents. 3. mI/Cr + PCr in the left ACC correlated positively with duration of metamphetamine use. | 1. GPC, PCh, Cr, PCr are known as the markers of glial density, and measure of cellular turnover, lower GPC + PCh/Cr + PCr revealed the occurrence of neurotoxic events in acute metamphetamine abstinents. 2. Lower concentrations of NAA/Cr + PCr, NAA + NAAG/Cr + PCr and GPC + PCh/Cr + PCr indicated compromised neuronal integrity and function as well as neurodegenerative or inflammatory processes initiated with acute metamphetamine abstinence. 3. Increased mI is related with increased proliferation rate of glial cells as well as activation of the innate immune response via microglia, astrocytes and endothelial cells. |
| 9. | Brody et al. 2017(42) | Smokers  (n = 30, 52.1 ± 8.1 yrs, 20% females)  Non-smokers  (n = 15, 47.6 ± 13.8 yrs, 26.7% females) | *PET scan* with  [^11^C]-DAA1106 | Cigarette smokers showed less [^11^C]-DAA1106 binding than non-smokers throughout the brain, indicating less TSPO availability. | Smoking results in global impairment of microglial activation. |
| 10. | Hillmer et al. 2017(44) | ADPs  (n = 15, 39.9 ± 9.7 yrs, 26.7% females)  HC  (n = 15, 35.0 ± 11.6 yrs, 26.7% females) | *PET scan* with [^11^C]-PBR28  *Endotoxin stimulation of peripheral monocytes* | 1. 10% lower TSPO levels in ADPs. 2. There is a negative association of TSPO levels in the hippocampus and striatum with alcohol dependence severity. 3. Peripheral monocyte response to lipopolysaccharide stimulation was lower in ADPs than HC for the pro-inflammatory cytokines IL-6 and IL-8. | Less activated microglia in the brain of ADPs and a blunted peripheral pro-inflammatory response. |
| 11. | Kalk et al. 2017(45) | ADPs  (n = 9, 45 ± 6yrs, 0% females)  HC  (n = 20, 45 ± 13 yrs, 30%) | *PET scan* with [^11^C]-PBR28 | 1. Lower [11C]-PBR28 V_T_, in the hippocampus in ADPs. 2. Hippocampal [^11^C]-PBR28 V_T_ was positively correlated with verbal memory performance in a combined group of HC and ADPs. 3. No evidence for increased microglial activation in ADPs. | 1. Lower glial density or an altered activation state with lower TSPO expression. 2. Abnormalities of glial function may contribute to cognitive impairment in ADP. |
| 12. | Frischknecht et al. 2017(63) | ADPs  (n = 39, 46.3 ± 11.4 yrs, 23% females)  HC  (n = 34, 44.5 ± 12.3 yrs, 21% females) | *In vivo ^1^H MRS* measurements within 24 hours after the last drink and after two weeks of abstinence | 1. Higher levels of markers of glutamatergic metabolism were associated with lower GM volumes in the hippocampus in early abstinence. 2. Over two weeks of abstinence, reduced NAA levels during intoxication persisted in patients with severe alcohol withdrawal symptoms. | 1. Reduced NAA levels are a consequence of ethanol rather than of withdrawal. 2. Severe alcohol withdrawal impairs NAA recovery. |
| 13. | Narendran et al. 2014(43) | Recently abstinent cocaine abusers  (n = 15, 39.9 ± 9.0 yrs, 53% females)  HC  (n = 17, 38.4 ± 8.1 yrs, 52.9% females) | *PET scan* with  [^11^C]-PBR28 | No significant differences in [^11^C]-PBR28 V_T_ was observed in the cortical and subcortical brain regions of cocaine abusers compared with HC. | The results do not support increased TSPO expression and, by extension, microglial activation in chronic cocaine-abusing humans. |
| 14. | Salo et al. 2011(61) | Short term methamphetamine abstinents  (n = 30, 36.3 ± 9.0 yrs, 50% females)  Long term methamphetamine abstinents  (n = 17, 36.3 ± 6.8 yrs, 76.5% females)  HC  (n = 24, 32.1 ± 7.9 yrs, 50% females) | *In vivo ^1^H MRS:* ACC, primary visual cortex  Estimates of premorbid intelligence, as assessed by NART | 1. Higher levels of ACC-Cho/NAA were seen short-term abstinent group compared to controls. 2. These metabolite levels did not differ between controls and the long-term abstinent group. 3. Lower ACC-NAA/Cr levels were observed in the short-term abstinents compared to controls and long-term abstinents. | 1. Elevated Cho levels might pertain to the following reasons: release of choline-containing compounds associated with acute damage to membrane, gliosis, and membrane biosynthesis. 2. Lower NAA levels might pertain to the ability of metamphetamine to disrupt mitochondrion function. 3. Longer remission periods are related to less membrane synthesis and turnover, which could be an explanation for the relative normal choline values in the brain regions of long-term abstinents. |
| 15. | Sailasuta et al. 2010(58) | Abstinent methamphetamine users  (n = 18, 35.0 ± 9.3 yrs, 38.9%)  HC  (n = 22, 31.9 ± 9.7 yrs, 45.5%) | *In vivo ^1^H MRS:* Frontal Region  Pre-morbid intelligence using the NART  Attention and psychomotor speed: Symbol Digit Modalities, the Grooved pegboard, and the Trail Making A tests  Cognitive flexibility and response inhibition: the Trail Making Test B and the Stroop Interference  Learning and memory: the Rey Auditory Verbal Learning Test (RAVLT) | 1. Elevated Glu and reduced NAA was seen in the frontal white matter region of abstinent methamphetamine users. 2. A significant correlation between low NAA levels in the frontal white matter and memory tasks was seen in the abstinent methamphetamine users. | 1. Possible explanations for elevated Glu in the frontal white matter could be: impairment of Glu receptors or Glu transporters in white matter; less conversion of Glu to Gln, or, less glutamatergic activity. 2. Reduced NAA levels might pertain to abnormalities in non-neuronal cells, microglia, and astrocytes, within the frontal white matter region of abstinent methamphetamine users. 3. Results also indicate the impact of long-term methamphetamine use on regional cerebral impairment in glutamate metabolism or in glutamatergic neurotransmission, which may lead to the persistent neurochemical abnormalities and cognitive deficits. |
| 16. | Sekine et al. 2008(84) | Abstinent methamphetamine users  (n = 12, 31.0 ± 3.5 yrs, 33%)  HC  (n = 12, 31.8 ± 3.4 yrs, 33%) | *PET scan* with ([^11^C](R)-PK11195) | 1. Mean levels of [^11^C](R)-PK11195 binding was higher in methamphetamine abusers than those in control subjects in the midbrain, striatum, thalamus, and orbitofrontal and insular cortices (>250% higher). 2. The binding levels of the radiotracer in brain regions, including midbrain, striatum, thalamus, and OFC and insular cortices correlated inversely with the duration of methamphetamine abstinence. | 1. Chronic self-administration of methamphetamine can cause reactive microgliosis in the brains of human methamphetamine abusers. 2. The activation level subsides over more extended periods of abstinence. |
| 17. | Salo et al. 2007(60) | Methamphetamine users  (n = 36, 36.9 ± 1.6 yrs, 63.9%)  HC  (n = 16, 32.2 ± 1.8 yrs, 50%) | *In vivo ^1^H MRS:* ACC, primary visual cortex  Estimates of premorbid intelligence, as assessed by NART  Stroop Attention Test | 1. The methamphetamine users exhibited reduced attentional control (i.e., increased Stroop interference) compared with the controls. 2. NAA/Cr and PCr levels were lower while Cho levels were elevated in ACC of methamphetamine users compared to controls. 3. ACC levels of NAA/Cr, but not of Cho/NAA, associated with attentional control in the methamphetamine users but not in controls. 4. No significant correlations were observed in the visual cortex of both groups. | 1. Long-term methamphetamine use disrupts the neuronal integrity within the ACC which is essential to regulate cognitive mechanisms that monitor conflict. 2. Moreover, medial frontal regions of the brain like ACC, are relatively rich in dopamine innervation which increases its vulnerability to the neurotoxic effects of long-term methamphetamine use. 3. Alterations in Cho levels seen in methamphetamine users may be implicated to a short-term pattern of response to neuronal injury instead of sustained neural changes that support cognitive regulation. |
| 18. | Sung et al. 2006(59) | Methamphetamine users:  with small cumulative methamphetamine dose  (n = 9, 35.3 ± 6.5 yrs, 33.3%)  with large cumulative methamphetamine dose (n = 21, 35.2 ± 3.9 yrs, 9.5%)  HC  (n = 20, 32.7 ± 6.2 yrs, 25%) | *In vivo ^1^H MRS:* frontal white matter and midfrontal gray matter | 1. Lower NAA levels in left frontal white matter of methamphetamine users. 2. There was no significant difference in the midfrontal gray matter NAA levels between the methamphetamine user groups, while the NAA levels did not associate with the cumulative methamphetamine dose and the duration of abstinence. 3. Greater myo-inositol levels in methamphetamine users. | 1. Methamphetamine-induces neurotoxic damage in gray matter may recover over time. 2. An increase in myo-inositol level may indicate a glial cell proliferation in response to the neuronal toxicity produced by methamphetamine. |
| 19. | Nordahl et al. 2005(57) | Methamphetamine users with sustained abstinence (1 year to 5 years)  (n = 8, 36.5 ± 2.8 yrs, 50%)  Recently abstinent methamphetamine users (1 month to 6 months)  (n = 16, 37.2 ± 2.3 yrs, 68.8%)  HC  (n = 13, 34.7 ± 2.3 yrs, 61.5%) | *In vivo ^1^H MRS:* ACC, primary visual cortex (gray matter) | 1. Methamphetamine users had abnormally Lower NAA/Cr levels was seen in ACC, in both recent and sustained methamphetamine abstinent groups. 2. Higher Cho/NAA levels in the ACC of recent methamphetamine abstinent cases during the first month of remission was seen. 3. No difference in NAA/Cr levels was observed in the primary visual cortex. | The findings were interpreted in the context of a reactive gliosis occurring in amphetamine users, which can lead to early elevations in Cho levels. As Cho is found in glial tissue, this might account for the relatively elevated Cho levels observed in subjects who were studied in the first month of remission. |
| 20. | Nordahl et al. 2002(56) | Methamphetamine Dependents  (n = 9, 32.5 ± 6.4 yrs, 0%)  HC  (n = 9, 32.7 ± 6.8yrs, 0%) | *In vivo ^1^H MRS:* anterior cingulum, DLPFW and VLPFW, primary visual cortex | 1. Low NAA/Cr levels were seen in the anterior cingulum of methamphetamine dependents. 2. Elevated Cho/Cr values in the anterior cingulum of methamphetamine dependents were seen compared to control subjects. | Low anterior cingulate NAA/Cr and high anterior cingulate Cho/Cr and cognitive deficits (attentional and response inhibition deficits) in methamphetamine dependents reflects neuronal loss or damage associated with neurotoxic effects of methamphetamine. |
| 21. | Ernst et al. 2000(55) | Abstinent methamphetamine users  (n = 26, 33.4 ± 7.9 yrs, 50%)  HC  (n = 24, 30.3 ± 4.6 yrs, 50%) | *In vivo ^1^H MRS:* midfrontal gray matter, right frontal white matter, and right basal ganglia | 1. N-acetyl aspartate was reduced by 6% in the frontal lobe and by 5% in the basal ganglia of methamphetamine users. 2. Choline and myo-inositol increased in frontal regions of methamphetamine users. | Decreased N-acetyl aspartate and increased myo-inositol in methamphetamine users most likely indicated glial proliferation in response to neuronal injury. |

**Note:** H^1^ MRS = proton Magnetic Spectrum Resonance; ELISA = Enzyme Linked Immunosorbent Assay; MRSI = Magnetic Resonance Spectroscopic Imaging; BDNF = Brain derived neurotrophic factor; IL-6 = Interleukin 6; CRP = C-reactive protein; TNF-α = Tumor necrosis factor- alpha; AUD = Alcohol Use Disorder; PET = positron emission tomography; TSPO = Translocator protein; ADP = Alcohol Dependent Patients; V_T_ = volume of distribution; DTI = Diffusion tensor imaging; Glu = glutamate; Cho = choline; mI = Myoinositol; Cr = Creatine; PCr = Phosphocreatine; NAA = N-acetylaspartate; FA = fractional anisotropy; ACC = anterior cingulate cortex, vACC = ventral anterior cingulate cortex; dACC = dorsal anterior cingulate cortex; PFC = prefrontal cortex; NAc = nucleus accumbens; AMY = amygdala; OFC = orbitofrontal cortex; DLPFW = dorsolateral prefrontal white matter; VLPFW = ventrolateral prefrontal white matter; NART = National Adult Reading Test; qPCR = quantitative polymerase chain reaction; mRNA = messenger RNA; HDAC2 = histone deacetylase 2; HDAC6 = histone deacetylase 6; HPP = hippocampus; CT = computed tomography; MDA = malondialdehyde; TBARS= thiobarbituric acid-reactive substance; FGF-23 = fibroblast growth factor; SUVs = standardized uptake values; TGF-β = transforming growth factor; GM = gray matter; SUV = standardized uptake values.
