# Supplementary material for: Inflammatory Markers in Substance Use and Mood Disorders: A Neuroimaging Perspective
Source: Front Psychiatry. 2022 Apr 26;13:863734. doi: 10.3389/fpsyt.2022.863734 (PMC9086785; doi:10.3389/fpsyt.2022.863734)
Supplement: Supplementary file 2 [file Table_2.docx]

**SUPPLEMENTARY TABLE 2** | Inflammation in Mood disorders

| **S/No.** | **Author**  **(year)** | **Number of participants (age, gender)** | **Methodology** | **Result** | **Interpretation** |
| --- | --- | --- | --- | --- | --- |
| 1. | Böttcher et al. 2020(108) | *Post-mortem tissue:*  Medicated MDD donors  (n= 6, Age and sex not reported)  Control donors with a history of depression  (n= 5, Age and sex not reported) | *Isolation of microglia* from the subventricular zone, thalamus, temporal lobe, and frontal lobe tissues  *Mass Cytometry*  *Flow Cytometry* | 1. No differences in the expression of HLA-DR, CD206, CD11c, CX3CR1. 2. Lower levels of the activation markers HLA-DR and CD6847 in MDD. 3. Microglia/macrophages expressed higher levels of the chemokine CD195 (CLL5), but comparable levels of IL-1β, IL-6, TNF, MIP- 1β (CCL4), IL-10, and MCP-1 (CCL2) between MDD and HC. 4. Increased levels of the homeostatic proteins P2Y12 receptor, TMEM119, and CCR5 (CD195) in microglia from all brain regions of individuals with MDD. | 1. Homeostatic functions may be enhanced in MDD microglia. 2. Increased expression of TMEM119 and P2Y12 in microglia clusters from MDD cases may reflect enhanced neuron-microglia communication via TGF-β1, which shows significant gene-environment interactions predicting adult depression in the context of early life trauma. 3. Expression of P2Y12 in microglia is essential for synaptic plasticity and adult hippocampal neurogenesis. |
| 2. | He et al. 2020(188) | MDD  (n = 44, 25.2 ± 1.4 yrs, 59% females)  HC  (n = 44, 25.6 ± 2.6 yrs, 45.5% females) | *ELISA* - serum IL-6, IL-8, IL-17A, MCP-1 and VEGF levels | MDD patients displayed an increased concentration of 13 pro-inflammatory and anti-inflammatory cytokines accompanied by one decreased cytokine in peripheral serum. | Cytokines were associated with depression, and the IL-6 and VEGF may serve as predictive biomarkers for depression. |
| 3. | Schlaaff et al. 2020(103) | *Brain tissues:*  Mood disorders  (n = 20, Median: 53.5 yrs, 45 % females)  HC  (n = 20, Median: 56.0 yrs, 45 % females) | *IHC* | 1. Elevated lymphocyte density was observed in 7/20 mood disorder patients, and in 1 of 20 HC. 2. There were different patterns of infiltration affecting cortical or subcortical white matter regions in some patients, while some presented diffuse infiltration. | Lymphocyte infiltration occurs in a more significant proportion of mood disorder patients than healthy controls indicating neuroinflammation associated with an impairment of the BBB in mood disorders. |
| 4. | Druzhkova et al.  2019(126) | MDD  (n = 33, 32.89 ± 7.82 yrs, 58% females)  HC  (n = 43, 30.51 ± 5.50 yrs, 56% females) | *Schulte tables stress tasks.*  *ELISA*  *Immunoassay system* | 1. The MDD group exhibited an elevated HPAA activity and IL-6 and CNTF levels at baseline. 2. Cortisol levels were increased in the MDD patients at baseline and 60 minutes after cognitive stress. 3. ACTH levels reduced significantly in MDD patients after the cognitive task. 4. A specific stress-induced increase in glucose and TNF-α was revealed in the MDD group, absent in HC. 5. There was a significant decrease of cortisol from baseline to 60 min after cognitive task in both patients and HC. | 1. TNF cytokine is involved in response to acute stress, and its answer is different in MDD and HC. 2. Excessive TNF release may be a marker of damaged immune mechanisms in mood disorders, and its assessment before and after a stress test may have potential diagnostic or prognostic value. 3. The increase in baseline cortisol and ACTH in the MDD group corresponds with chronic HPAA hyperactivity, which may be linked to lowered parasympathetic influence. 4. The reaction of simple metabolic and pro-inflammatory indices to a mild stressogenic challenge may indicate a depressive state. |
| 5. | Scaini et al. 2019(92) | BD  (n = 31, 36.9 ± 12.0 yrs, 77.4% females)  HC  (n = 25, 37.0 ± 10.2 yrs, 72% females) | *Gene expression*  *Immunoblotting* | 1. BD patients revealed lower levels of Parkin, p62/SQSTM1, and LC3A and overexpression of TSPO pathway proteins (TSPO and VDAC) in terms of mRNA and protein levels. 2. There was a negative correlation between mitophagy-related proteins and TSPO levels, while VDAC correlated negatively with p62/SQSTM1 and LC3 protein levels. 3. The genetic expression of NLRP3-related proteins like NLRP3, ASC, and procasp1 was found to be upregulated in BD patients, followed by an increase in caspase-1 activity as well as IL-1β and IL-18 levels. 4. A strong positive association between NLRP3-related inflammasome activation and TSPO-related proteins was seen. | 1. TSPO-VDAC complex upregulation in BD patients. 2. The simultaneous downregulation of mitophagy proteins and NLRP3 inflammasome activation could accumulate dysfunctional mitochondria, resulting in inflammation and apoptosis.   Mitochondrial dysfunction assessed in peripheral blood is associated with BD. |
| 6. | Mora et al.  2019(118) | Manic patients  (n=32, 41.25 ± 12.9 yrs, 43.7% females)  Euthymic Bipolar patients  (n=52, 47.52 ± 11.9 yrs, 50% females)  HC  (n = 49, 48.3 ± 12.1yrs, 57.1% females) | *ELISA* - Serum BDNF measurement  *Neuropsychological assessment* | 1. BDNF levels were decreased in euthymic and manic individuals. 2. Inflammatory and oxidative stress measures were increased in bipolar individuals compared to HC. 3. BDNF levels were associated with executive functioning, verbal memory, and other demographic variables. 4. Neither inflammatory markers, oxidative stress markers, nor other relevant clinical variables are associated with cognitive outcomes. | 1. Of all the peripheral neurobiological factors analyzed, BDNF was the only one significantly associated with cognitive dysfunction in BD individuals. 2. This study emphasizes the role of BDNF not only across mood phases but also in cognitive functioning. 3. The presence of verbal memory impairment across mood phases may indicate that these deficits are trait markers of BD. 4. BDNF levels may not reflect the high genetic risk for BD, acting as a state marker rather than a trait marker for the disease. |
| 7. | Uint et al.  2019(148) | RD  (n = 34, 50.5 ± 12.0 yrs, 85.3% females)  BAD  (n = 43, 46.5 ± 10.6 yrs, 88.4% females)  HC  (n = 41, 50.4 ± 9.0 yrs, 80.5% females) | BDNF  *Turbidimetric immunoassay* | Although no differences were found in gene expression, BDNF and IL-1β plasma concentration was increased in the RD group, even with antidepressant treatment. | 1. The increase of BDNF and IL-1β suggests that these drugs have no effect on IL-1β secretion and that the inflammasome may play a role in therapy response. 2. Taken together, both BDNF and IL-1β plasma concentrations could be used for the early identification of RD patients. |
| 8. | Sneeboer et al 2019(95) | Paraffin (control n = 12, 75.6 ± 11.8 yrs, 41.7% females; BD n = 16,72.0 ± 10.4 yrs, 25% females)  Snap frozen  (control n = 16, 75.1 ± 13.0 yrs, 73.3% females; BD n = 15, 74.7 ± 8.0 yrs, 20% females)  **Isolated Microglia**:  *mRNA expression*  (control n = 16, 79.7 ± 11.9 yrs, 68.7% females; BD n = 12, 74.9 ± 11.1 yrs, 58.3% females)  *Protein expression*  (control n = 17, 83.1 ± 10.4 yrs, 64.7% females; BD n = 9, 73.2 ± 17.3 yrs, 66.7% females)  *LPS response*  (control n = 19, 79.4 ± 11.8 yrs, 68.4% females; BD n = 9, 73.6 ± 19.7 yrs, 66.7% females) | *Immunostaining*  *Immunofluorescent staining*  *Microglial isolation*  *Flow cytometry*  *RNA extraction*  *cDNA synthesis*  *qPCR* | 1. Similar microglial density was seen in BD and controls. 2. No differences in expression between patients and controls for microglial and immune-activated genes. 3. No differences in the expression of genes involved in proinflammatory signaling (IL1B and IL6), genes related to anti-inflammatory functioning (CD163 and MRC1), TMEM119, a microglial specific gene known for its homeostatic properties and CX3CR1, a gene related to microglial migration and neuron-glia interaction in the MFG, STG, and thalamus. 4. No differences in protein expression, including CD16 and CX3CR1, between controls and BD in the MGF and thalamus. 5. Microglia from BD and controls both responded to LPS with increased expression of IL1B, IL6, and TNF. The effect was not significantly different between microglia of patients with BD and controls. The same was observed in microglia from the STG and thalamus. | The results do not support microglial immune activation, since differences in microglial density, and the expression of mRNA levels genes related to microglial function in fixed and frozen brain tissue were not found. |
| 9. | Kuwano et al. 2018(88) | Drug-free MDD patients  (n = 34, 30.5 ± 7.0 yrs, 41.2% females)  HC  (n = 34, 30.5 ± 7.0 yrs, 41.2 % females) | *Biotinylation of antibodies*  *ELISA* | 1. Most neuron-related blood biomarkers had a moderately to strongly positive correlation with CD81 (NDE). 2. IL34/CD81 levels were significantly higher in the MDD group compared to the HC group. 3. SYP, SYP/CD81, and TNFR1/CD81 were positively correlated with severe depression and/or various sub-symptoms. | 1. Not only SYP and TNFR1 but also IL34 are important blood biomarkers for patients with MDD. 2. Some neuron-related blood biomarkers have been revealed to positively correlate with CD81 (NDE), suggesting that these substances may be carried via exosomes in the brain. 3. Higher IL34/CD81 levels in patients with MDD have suggested neuronal damage and microglial dysfunction linked to the pathophysiology of MDD, at least during the acute phase. 4. OXR/CD81 levels have exhibited a higher trend in patients with MDD, suggesting that OXR in the brain may be upregulated in MDD. |
| 10. | Vasconcelos-Moreno et al. 2017(89) | BD - I  (n =36, 47.2 ± 10.1 yrs, 75% females)  Siblings  (n = 39, 49.7 ± 14.4 yrs, 69% females)  HC  (n = 44, 45.8 ± 12.1 yrs, 54.5% females) | *ELISA* - Oxidative stress parameters including GPx, GR, GST, and 3-NT  *Flow cytometry* - IL-6, IL-10, and TNF-α concentrations  *qPCR* | 1. Levels of both IL-6 and IL-10 were significantly higher in patients than in HC. 2. C-C motif chemokine 11 levels were increased in siblings compared with HC, and a similar tendency was found in patients compared with HC. 3. GPx activity was decreased in patients compared with HC and siblings. 4. No differences in BDNF levels between the BD-I, sibling, and HC groups. | 1. BD showed higher levels of inflammatory and oxidative markers. 2. Greater levels of oxidative lipid damage seen indicate stimulation of inflammatory activation due to increased oxidative stress in patients. |
| 11. | Doolin et al. 2017(130) | MDD  (n = 57, 28.3 ± 12.1 yrs, 35% females)  HC  (n = 40, 27.5 ± 5.6 yrs, 32.5% females) | *LC-MS* – to assess cortisol and cortisone levels.  *qPCR* - mRNA expression of HSD11β-1, IL-1β, IFN-γ, and TNF-α | 1. No differences in salivary cortisone concentrations between depressed patients and HC. 2. There were no differences in whole blood HSD11β-1 mRNA expression between depressed and HC. 3. Expression of HSD11β-1 was significantly positively correlated with average morning cortisone and peak cortisone concentration. 4. Significantly higher expression of IL-1β mRNA in depressed patients compared to HC. 5. No significant difference was noticed in mRNA expressions of IFN-γ, IL-6, or TNF-α between depressed patients and HC. | 1. A hyperactive stress system in depressed patients. 2. The increased severity of depressive symptoms exacerbates HPAA dysregulation. 3. No differences in cortisone concentrations between depressed patients and healthy controls indicate that the disruption of the HPAA witnessed in depressed patients is occurring as an increase of cortisol while maintaining similar cortisone levels. 4. The association of cortisol within the depressed group to whole blood mRNA expression of IL-1β is indicative of a link between HPA and immune dysregulation in depression. |
| 12. | van den Ameele et al. 2017(143) | BD on acute mood episode:  Depressive  (n = 35, 43.7 ± 9.7 yrs, 68.6% females)  Hypomanic  (n = 32, 42.9 ± 12.7 yrs, 46.9% females)  HC  (n = 29, 43.0 ± 11.4 yrs, 53.3% females) | *Mood symptom severity assessment:*  HDRS-17  YMRS  *ECL immunoassay technique* - baseline and after two months | 1. No significant differences in neurotrophic markers between patients and HC. 2. Significantly increased TNF-α levels in patients and a subsequent normalization during euthymia. 3. None of the biomarkers strongly correlated to mood symptom severity. | 1. Findings do not suggest mood state-related alterations in neurotrophic markers. 2. Weak evidence for mood state-related TNF-α levels. |
| 13. | Brisch et al 2017(96) | Brain Controls (n = 22, median age: 52 yrs, 68% females)  Suicidal (n = 24, median age: 48 yrs, 54% females)  Non-suicidal n = 21, median age: 54 yrs, 47% females) | *Microglial immunostaining* | 1. No significant differences in microglia density between all suicidal and non-suicidal patients and controls by means of the analysis of rostral and caudal subregions of the DRN and the cumulative analysis of all DRN subnuclei. 2. Only non-suicidal depressed patients revealed significantly decreased microglial reaction versus both suicidal depressed patients and controls in the cumulative analysis of the DRN. 3. The microglia density in the entire affective disorders group revealed significant correlation with the AgNOR number 4. No associations between microglia density and AgNOR parameters were found in controls. | 1. Increased microglia activity observed in the DRN of depressed suicides versus non-suicides reveals that activated microglia may induce oxidative stress in target neurons. 2. Microglia may exert either devastating or restoring effect on neuronal function, which is related to the prevalence of damaging or supportive subpopulations in activated microglia. 3. The observed microglia increase in depressed suicides compared to non-suicides may reflect an attempt for the restoration of decreased neuronal plasticity. 4. Therefore, the decreased microglial reaction in the non-suicidal depressed subgroup might rather be interpreted as a suicide-preventive effect. 5. The results suggest a possible suicide-preventive effect of microglial reaction restricted to this subgroup, whereas an opposite effect may exist in depressed suicidal patients. |
| 14. | Tatay-Manteiga et al. 2017(110) | Early-Stage BD  (n = 25, 43.4 ± 10.3 yrs, 52% females)  Late-Stage BD  (n = 23, 45.1 ± 9.8 yrs, 52.2% females)  Healthy Siblings  (n = 23, 41.5 ± 11.8 yrs, 69.6% females)  HC  (n = 21, 36.7 ± 10.9 yrs, 66.7% females) | *ELISA* - plasma concentration of BDNF and serum concentration of TNF-α, IL-6, IL-10, NT-3 | 1. IL-10 concentration was significantly increased in early-stage patients, healthy siblings, and controls compared to late-stage patients. 2. TNF-α concentration was significantly increased in late-stage patients compared to controls. 3. Total leukocytes, neutrophils, and monocyte count were significantly increased in late-stage patients compared to healthy siblings and controls. | A link exists between peripheral inflammation and different stages in BD. |
| 15. | Wang et al. 2016(141) | BD-I  (n = 234, 33.6 ± 11.7 yrs, 50.9% females)    BD-II  (n = 260, 31.6 ± 12.3 yrs, 48.1% females)    SBP  (n = 243, 33.0 ± 12.6 yrs, 58.8% females)  HC  (n = 140, 31.9 ± 8.2 yrs, 42.1% females) | *Antibody pair assay system* - TNF-α, CRP, TGF-β1, IL-8, and BDNF were measured. | 1. TNF-α, TGF-β1, and IL-8 were significantly higher in all BD patients than HC. 2. BD-I patients had significantly higher IL-8 levels than did BD-II and SBP. | 1. The immunological disturbance along the bipolar spectrum was most severe in BD-I. 2. No difference in biological markers levels was seen between SBP and BD-II patients. |
| 16. | Bogerts et al. 2016(102) | *Brain tissues:*  Mood disorders  (n = 18, 53.3 ± 11.2 yrs, 44.4 % females)  HC  (n = 20, 55.8 ± 8.8 yrs, 45 % females) | *IHC* | 1. 40 to 70 percent of the mood disorder patients had a moderately increased CD3-T-lymphocytes in mesiotemporal, diencephalon, frontal and temporal cortex, cingulate gyrus white matter. 2. B-Lymphocytes (CD20-cells) were only significantly increased in mood disorder patients' hippocampal/parahippocampal region. | The results indicate a possible role of neuroinflammation in the pathogenesis of mood disorders. |
| 17. | Cinar et al. 2016(144) | Male drug-free BD patients during manic and remission periods (n = 20, 31.8 ± 8.2 yrs)  Male HC (n = 20, 32.3 ± 7.5 yrs) | *qRT-PCR* - gene expression levels of BDNF, tPA, GR, HSP-70, and TNF-α | 1. Reduced expressions of BDNF and tPA mRNA in mania compared to controls. 2. BNDF and tPA mRNA levels increased in remission, but they were still lower than those of the controls. 3. Only BDNF mRNA expression varied between mania and remission periods. | 1. BDNF and tPA may be biomarkers of BD. 2. Proteolytic conversion of BDNF may be necessary for the pathophysiology of BD. 3. The change in BDNF levels between mania and remission could be adaptive and used to follow the progression of BD. |
| 18. | Becking et al. 2015(131) | Depressed men:  No lifetime hypomanic episode (n = 209, 43.9 ± 11.6 yrs)  Lifetime hypomanic episode (n = 56, 42.7 ± 11.0 yrs)  Depressed women: No lifetime hypomanic episode (n = 431, 40.4 ± 12.4 yrs)  Lifetime hypomanic episode (n = 68, 38.3 ± 11.9 yrs) | *Composite International Diagnostic Interview (CIDI)*  *Salivettes to sample baseline cortisol*  *Cortisol Awakening Response*  *ELISA  Baseline inflammatory markers included CRP, IL-6, and TNF-α*  *International Physical Activity Questionnaire*  *Women’s Health Initiative Insomnia Rating Scale* | 1. In depressed men and women, none of the cortisol indicators and inflammatory markers were independently associated with a hypomanic episode. 2. Effect modification was found of diurnal cortisol slope and CRP in relation to a hypomanic episode. 3. Depressed men with high levels of diurnal cortisol slope and CRP had an increased odds of having a hypomanic episode. However, no significant differences were found in women. | The combination of high diurnal cortisol slope and high CRP may differentiate between unipolar and bipolar disorder. This stresses the importance of considering HPA-axis and immunological activity simultaneously. |
| 19. | Isgren et al. 2014(94) | BD  (n = 121, median: 36.0 yrs, 61.2% females)  HC  (n = 71, median: 32.0, 63.4% females) | Cytokine measurement: *Single plex assay* – IL6  *MSD 96-well multi-array and multi-spot human cytokine assay* – Other Cytokines  *Immunonephelometry* – CSF/serum albumin ratio measurement | 1. IL-8 levels were higher in BD patients when compared to HC. 2. Within the patient group, IL-8 concentrations were positively associated with CSF/serum albumin ratio, lithium and antipsychotic treatment. 3. Patients with lithium- and/or antipsychotic medication had significantly higher IL-8 concentrations compared to patients without such medication and compared to HC. | The findings might reflect immune aberrations in BD because of medication. |
| 20. | Spanemberg et al.  2014(106) | Depressed non-melancholic  (n = 20, 48.4 ± 7.7 yrs, 90% females)  Depressed melancholic  (n = 13, 52.8 ± 10.7 yrs, 76.9% females)  HC  (n = 54, 47.4 ± 9.97 yrs, 74.1% females) | BDNF measurement of serum concentrations  *TBARS assay* | 1) Depressed patients had significantly higher levels than HC on oxidative stress markers; PCC, immunity markers; IL-4, and IL-6 variables.  2) Melancholic patients generated lower IFN-γ (compared with non-melancholic depressed patients) and TBARS (compared with both the non-melancholic subset and controls) and returned higher IL-6 levels than HC.  3) Both depressive groups generated higher PCC scores than HC, with no difference between melancholic and non-melancholic subsets. | 1. Melancholic patients scored lower across lipid oxidative stress markers (versus non-melancholic patients) and immunological markers (versus both controls and non-melancholic patients). 2. Melancholic depression may constitute specific dysregulation in the balance of pro-inflammatory/anti-inflammatory and Th1/Th2 cytokines, with a sustained increase in hypercortisolemia and catecholamines that then upregulate IL-6 levels. 3. The inability of PCC to discriminate depressive subgroups may be more a reflection of "depression" than of depressive "subtype." |
| 21. | Hercher et al. 2014(97) | Brain Controls (n = 20, age 45.3 ± 6.5 yrs,  30% females)  Schizophrenia (n = 20, age 44.7 ± 6.9 yrs, 35% females)  BD (n = 20, age: 47.4 ± 0.7 yrs, 60% females) | *Immunohistochemistry: bilateral MFG, rostral to the genu of the corpus callosum*  *Microscopic image analysis*  *Area Fraction*  *Spatial pattern analysis*  *ELISA*  *Immunoblotting* | 1. CNPase levels with protein expression higher in the left hemisphere in all groups. 2. A significant effect of diagnosis on oligodendrocyte density, reflecting increased oligodendrocyte density in the BD group compared with the control group. 3. The oligodendrocyte nuclear area and diameter did not differ among the groups. 4. Decreased GFAP area fraction in BD compared with the control group. 5. Higher astrocyte CC in BD than the control group. 6. Astrocyte densities differed significantly among the groups. 7. Oligodendrocyte CC also differed among the groups, with the suicide group showing higher oligodendrocyte CCs than the control group and the psychiatric nonsuicide group. 8. BD patients on prescribed mood stabilizers showed a trend toward increased oligodendrocyte density and increased MBP immunoreactivity relative to those who had not been prescribed mood stabilizer. | 1. CNPase is present in oligodendrocyte cell bodies and is frequently used as a marker for this cell population. 2. The increase in oligodendrocytes may not be accompanied by higher white matter myelin concentration. 3. The results suggest that mood stabilizers, alcohol use and illicit drug use may influence oligodendrocyte density in individuals with BD. 4. Increased astrocyte clustering could result in disruption of structural support of axons in BD. |
| 22. | Dean et al 2013(98) | Control Group A  (n = 20, age 47 ± 4.1 yrs, 20% females)  Control Group B  (n = 10, age 56 ± 4.8 yrs, 30% females)  Control Group C  (n = 10, age 63 ± 4.1 yrs, 40% females)  MDD  (n = 10, age 61 ± 5.4 1 yrs, 40% females)  BD  (n = 10, age 60 ± 4.0 yrs, 40% females)  Schizophrenia  (n = 19, age 48 ± 4.4 yrs, 21% females) | *Western blot analysis*  *RNA extraction*  *PCR* | 1. Levels of transmembrane TNF-α were increased in ventral ACC, but not dlPFC, in subjects with BD. 2. Levels of soluble TNF-α did not vary significantly with diagnosis in cortex from subjects with mood disorders. 3. No increase in CD11b in the cortex of people with mood disorders. 4. Levels of TNF mRNA did not vary with diagnosis in either ventral ACC or, dlPFC in people with mood disorders versus controls. 5. Levels of TNFR1 mRNA did not vary with diagnosis in cortex from subjects with mood disorders. 6. Significant variation in levels of TNFR2 mRNA with diagnoses in dlPFC, but not ventral ACC, in mood disorder individuals. | 1. There are complex changes in TNF-α related pathways results from increased proinflammatory activity in the cortex of people with mood disorders. 2. Increased levels of tmTNF in ventral ACC in BD and in frontal region in MDD possibly relates to increased reverse signaling by that protein in these cortical regions. 3. The changes in TNFR2 mRNA in dlPFC from both MDD and BD indicates change in bidirectional signaling1 in dlPFC. 4. Since dlPFC is known to be critical in maintaining cognitive processes, the changes in TNF-α related pathways could be involved in cognitive changes associated with MDD and BD. 5. ACC is important in controlling mood and therefore changes in TNF-α related pathways could be involved in changes in mood, particularly in BD. |
| 23. | Uddin et al. 2011(90) | Depressed  (n = 33, 43.5 ± 11.9 yrs, 69.7% females)  Non-depressed  (n = 67, 46.2 ± 18.7 yrs, 55.2% females) | *Methylation microarrays*  *Bioinformatic functional analyses* | 1. There are genome-wide differences in methylation profiles among individuals with and without lifetime depression in a community-based setting. 2. Genes uniquely unmethylated in each of the two groups show evidence for the involvement of inflammatory-related pathways and processes previously implicated in depression. The functional significance of these results demonstrates elevated levels of two inflammatory markers, IL-6 and CRP, among those with lifetime depression. 3. There is an inverse correlation between methylation of IL-6 CpG and circulating IL-6 and CRP levels among those with lifetime depression.   Among individuals with lifetime depression, there was a signal of lipoprotein-related functions in the uniquely unmethylated gene set. | 1. Depression has been characterized as an immune response directed against disrupted lipid membrane components and by-products of lipid peroxidation. 2. The etiology of depression involves dysregulated biologic processes that are altered across multiple levels of the organization, from molecular-level changes in methylation profiles observed in blood-derived DNA to systemic changes in pro-inflammatory cytokine signaling pathways that, although kept in the periphery, are known to be capable of affecting neurogenesis in the CNS and are related to disruptions in neuroplasticity in mood relevant brain regions. |
| 24. | Kaestner et al. 2005(133) | MMD  (n = 21, 51.4 ± 12.0 yrs, 71.4 % females)  NMMD  (n = 16, 36.8 ± 12.4 yrs, 68.8 % females)  HC  (n = 37, 44.6 ± 13.9 yrs, 70.2% females) | *ELISA* | 1. Increased IL-1h production upon mitogen stimulation (PHA) and decreased IL-1RA/IL-1h in acutely depressed patients with NMMD compared to HC, to acutely depressed patients with MMD and to NMMD patients in remission. 2. Plasma ACTH concentration is increased in patients with acute and remitted MMD compared to HC. 3. Serum cortisol concentration increases in patients with acute MMD compared to remitted stage and HC. | 1. Signs of activation of the HPAA in MMD. 2. IL-1RA/IL-1β ratio was initially decreased before significant elevation with clinical improvement. It was caused by the antidepressant treatment leading ultimately to the normalization of IL-1β production. 3. Lowered IL-1RA/IL-1β ratio in acute NMMD probably indicates an inflammatory response in NMMD, especially with the total elevated cytokine production. 4. In MMD, the inflammatory response might be alleviated by activating the HPAA. |
| 25. | Chen et al. 2020(135) | BD-I  (n = 22, 28.1 ± 12.0 yrs, 82% females)  MDD-I  (n = 22, 28.7 ± 13.3 yrs, 82% females)  HC  (n = 22, 27.4 ± 10.2 yrs, 82% females) | *WCST* - Executive function assessment  *ELISA*  *VBM* | 1. BD patients exhibited higher levels of TNFR1, a greater number of deficits in WCST, and smaller GM volume in the MFC compared with MDD and HC. 2. Positive associations were observed between the MFC volume, executive function, and the TNFR1 level. | 1. GM volume reduction in the MFC, a greater level of systemic inflammation, and executive dysfunction BD-I patients. 2. A divergent effect of brain and systemic inflammation functioning in the early phase (first episode) of affective disorder. |
| 26. | Huang et al. 2018(152) | MDD (n = 34):  TRD (n = 20; 45.1 ± 10.2 yrs, 60% females)  non-TRD  (n = 14; 47.9 ± 11.1 yrs, 57% females)  Healthy controls  (n = 34, 42.5 ± 8.5 yrs, 58.8% females) | *PET scan* with ^8^F-FDG | 1. Elevated serum levels of TNF-α R1 were seen in TRD patients than the healthy control or non-TRD patients. 2. In the MDD group, higher serum concentrations of TNF-α R1 significantly correlated with decreased SUV in ACC and bilateral CN, more consistent in the TRD group. | Increased TNF-α R1 was associated with impaired CN and ACC glutamatergic neurotransmission in MDD patients, particularly in the TRD. |
| 27. | Li et al.  2018(124) | MDD  (n = 50, 28.7 ± 9.0, 50% females)  HC  (n = 30, 27.4 ± 9.0 yrs, 50% females) | *PET scan* with [18F]-FEPPA  *T-1 weighted MRI*  *TSPO V_T_ analysis*  *RBANS* | 1. Patients with MDD showed elevated TSPO V_T_ in all regions of interest (white matter, grey matter, frontal cortex, temporal cortex, and hippocampus). They were impaired on the attention and delayed memory domains of the RBANS. 2. In the frontal cortex, increased TSPO V_T_ was associated with lower scores on the RBANS attention domain when the analysis was corrected for age, gender, education, and depressive symptoms. | 1. Depression-associated pathophysiological processes, mediated by microglia in the frontal cortex, significantly contribute to cognitive deficits. 2. Microglial pathology may primarily contribute to cognitive dysfunctions. |
| 28. | Swartz et al.  2017(104) | Healthy participants  (n = 174, 8-22 years, 60% females) | *Functional MRI -* Emotional face matching task (Fearful and Angry Faces > Shapes)  *CRP Assay* | 1. A significant interaction was found between CRP and sex: in men, but not women. 2. Higher CRP was associated with higher threat-related AMY activity. 3. Effects were observed in the right AMY when analyzing the entire participants and the subset without psychiatric diagnoses. In contrast, outcomes for the left AMY were only observed for the sample subset without psychiatric diagnoses. | 1. The positive association between CRP levels and AMY activity may be a risk pathway that plays a promising role in the etiology of internalizing problems in men compared to women. 2. Inflammation may be more strongly associated with AMY activity to clear rather than more ambiguous threat signals. 3. More robust effects for the right AMY may involve laterality differences in habituation. |
| 29. | Haarman et al.  2015(51) | BD  [n = 22, 44.5 (24–61), 55% females]  HC  [n = 24, 38.5 (19–67), 50% females) | *PET scan* with [^11^C]-(R)-PK11195  *In vivo proton (^1^H) MRS* | 1. Hippocampal volumes of patients were similar to HC after correcting individual whole-brain volume variations. 2. There was a decrease in NAA + NAAG and Cr + PCr levels in the left hippocampus of BD-I patients suggesting a decreased neuronal integrity in this region. 3. Several associations were seen: positive - microglial activation with the NAA + NAAG concentration and the depression score, alcohol use with NAA + NAAG concentration; negative - between Cr + PCr concentration and experienced occupational disability. 4. Duration of illness is associated positively with brain volume. |  |
| 30. | Setiawan et al. 2015(91) | MDD <10 yrs untreated  (n = 25, 31.8 ± 10.3 yrs, 64.0% females)  MDD ≥ 10 yrs untreated  (n = 25, 37.1 ± 11.0 yrs, 60.0% females)  HC  (n = 20, 33.6 ± 12.8 yrs, 55.0% females) | *PET scan* with FEPPA  *2D axial proton-density MRI*  *DNA Extraction-Binding affinity*  *Polymorphism Genotyping* | 1) Elevated TSPO in MDE in all brain regions compared with the controls (main effect of diagnosis.  2) MDE patients had significantly greater TSPO V_T_ in the PFC, ACC, and insula compared with healthy controls after controlling for genotype effect.  3) In both groups, the effect of the rs6971polymorphism was in which individuals with high-affinity binding had greater TSPO compared with individuals with mixed-affinity binding.  4) Differences in TSPO between the patients with MDE and healthy controls remained significant if age was applied as a covariate.  5) The total HDRS score was positively correlated with TSPO in the ACC after correcting for the rs6971 genotype.  6) In Patients with MDE, none of the serum markers of inflammation had a significant positive correlation with TSPO in the primary regions of interest. | 1. TSPO is upregulated in activated microglia, and elevated TSPO implies that greater microglial activation, a potentially targetable process of neuroinflammation, is present during MDE. 2. MDE associated with elevated TSPO across all brain regions examined. 3. Greater TSPO in specific regions and/or their associated circuitry may influence the expression of symptoms within MDE. |
| 31. | Haarman et al. 2014(99) | BD-I (n = 14, 46.2 ± 10.6 yrs, 50% females)  HC (n =11, 41.1 ± 18.3 yrs, 70% females) | *PET scan with [11C]-(R)-PK11195*  *MOODINFLAME*  *Inventory of Depressive Symptoms – Clinician Version (IDS-C30)*  *Young Mania Rating Scale (YMRS)*  *MRI* | A significantly increased [11C]-(R)-PK11195 binding potential, which is indicative of neuroinflammation, was found in the right hippocampus of the patients when compared to the healthy controls. | 1. A possible differential [11C]-(R)-PK11195 binding potential between the right hippocampus and the left dlPFC could also be due to aberrant connectivity between the PFC regions and limbic system regions in BD. 2. Focal neuroinflammation in the right hippocampus of BD-I patients may pertain to in vivo immune activation involved in the pathophysiology of BD. |
| 32. | Frodl et al.  2012(134) | MDD  (n = 40, 41.4 ± 10.9 yrs, 60% females)  HC  (n = 43, 37.0 ± 13.7 yrs, 58.1% females) | *MRI T1 data acquisition.*  *ELISA*  *PCR* | 1. Patients with MDD showed smaller hippocampal volumes and increased inflammatory proteins IL-6 and CRP compared with controls. 2. Childhood maltreatment was associated with increased CRP. 3. MDD patients with minor glucocorticoid-inducible GILZ or SGK-1 had smaller hippocampal volumes. 4. There is a strong positive effect of GILZ and SGK-1 mRNA expression and further inverse effects of IL-6 concentration on hippocampal volumes. | 1. Childhood maltreatment, peripheral inflammatory, glucocorticoid markers, and hippocampal volume are interrelated factors in the pathophysiology of MDD. 2. Glucocorticoid-inducible genes GILZ and SGK-1 might be promising candidate markers for hippocampal volume changes relevant for diseases like MDD. |

**Note:** BD = bipolar disorder; BD-I = bipolar disorder type I; SBP = short-duration hypomania; BD-II = bipolar disorder type II; HC = healthy controls; MDD= major depressive disorder; RD = resistant depression; BAD = bipolar affective disorder; MMD = melancholic major depression; NMMD = non-melancholic major depression; TRD = treatment resistant disorder; NTRD = non-treatment resistant disorder; MDE = major depressive episode; ELISA = enzyme-linked immunosorbent assay; IHC = immunohistochemistry; LPS = lipopolysaccharide; GPx = glutathione peroxidase; GR = glutathione reductase; GST = glutathione S-transferase; 3-NT = 3-nitrotyrosine; IL-6 = interleukin-6; IL-8 = interleukin-8; IL-10 = interleukin-10; TNF-α = tumor necrosis factor alpha; BDNF = brain derived neurotrophic factor; TGF= transforming growth factor; CRP = c-reactive protein; IFN-γ = interferon gamma; HLA-DR = human leukocyte antigen – DR isotype; NT-3 = neurotrophin-3; HPAA = hypothalamic-pituitary-adrenal Axis; CD = cluster of differentiation; MIP = macrophage inflammatory protein; PCC = protein carbonyl content; CNTF = ciliary neurotrophic factor; NLR3 = NOD-like receptor proteins; ADC = apoptosis-associated speck-like protein containing CARD; NDE = neuronally derived exosomes; SYP = synaptophysin; TNFR1 = tumor necrosis factor receptor 1; OXR = oxidation resistance; GILZ = glucocorticoid-induced leucine zipper; SGK-1= serum-glucocorticoid-regulated kinase-1; CSF = cerebrospinal fluid; TMEM = transmembrane protein; TBARS = thiobarbituric acid reactive substances; NAA = N-acetylaspartate; NAAG = N-acetyl-aspartyl-glutamate; Cr = creatine; PCr = phosphocreatine; CNS = central nervous system; BBB = blood brain barrier; dlPFC = dorsolateral prefrontal cortex; tPA = tissue plasminogen activator; GR = glucocorticoid receptor; HSP-70 = heat shock protein; qPCR = quantitative polymerase chain reaction; LC-MS = liquid chromatography-mass spectrometry; MFG = middle frontal gyrus; STG = superior temporal gyrus; MFC = middle frontal cortex; CN = caudate nucleus; ACC = anterior cingulate cortex; PFC = prefrontal cortex; AMY = amygdala; WCST = wisconsin card sorting test; HDRS-17 = hamilton depression rating scaling; YMRS = young mania rating scale; RBANS = repeatable battery for the assessment of neuropsychological status; ECL = electrochemiluminescence; TSPO = translocator protein; VDAC = voltage-dependent anion channels; MRS = Magnetic resonance spectroscopy; PET = positron emission tomography; V_T_ = distribution volume; SUV = standardized uptake values; GM = gray matter; VBM = voxel based morphometry; DRN = dorsal raphe nucleus; AgNOR = argyrophilic nucleolar organising region; IBA -1 = ionized calcium-binding adaptor molecule-1; GFAP = glial fibrillary acidic protein; CNPase = 2,3-cyclic-nucleotide 3-phosphodiesterase; CC = coefficient of clustering; MBP = myelin basic protein; TNFR1 = tumor necrosis factor receptor 1; TNFR2 = tumor necrosis factor receptor 1; Sz = schizophrenia.
